# TRACE-DDI: A Hybrid Framework of Transformer–GAT Context Encoder and Pathway-Anchored Knowledge Graphs for DDI Prediction

**DOI:** 10.34133/csbj.0057

**Published:** 2026-05-15

**Authors:** Junku Kim, Taehyeok Seo, Kyuri Jo

**Affiliations:** ^1^ Department of Computer Engineering, Chungbuk National University, Cheongju, Republic of Korea.; ^2^ Department of Statistics, Chungbuk National University, Cheongju, Republic of Korea.

## Abstract

Accurate prediction of drug–drug interactions (DDIs) is crucial for preventing adverse drug reactions and improving drug development efficiency. However, most existing DDI prediction models treat chemical structure and biological knowledge separately, failing to capture multiscale biological context. We present TRACE-DDI, a hybrid framework that integrates 3 complementary modalities within a single end-to-end architecture: (a) the Simplified Molecular Input Line Entry System (SMILES)-based chemical sequences, (b) molecular graph topology, and (c) pathway-anchored biological knowledge graphs. TRACE-DDI employs a Transformer-Graph Attention Network (GAT) cross-modal context encoder that performs multilayer graph-based feature propagation across heterogeneous biological representations, enabling both fine-grained chemical understanding and global pathway-level context aggregation. Experimental results on the benchmark dataset demonstrate that TRACE-DDI consistently outperforms state-of-the-art baselines across all major evaluation metrics. Beyond predictive accuracy, TRACE-DDI provides biologically interpretable insights: pathway-level subgraph visualization and centrality analysis prioritize shared pathway nodes that occupy prominent topological positions in merged drug subgraphs, thereby providing hypothesis-generating biological context for predicted DDIs. Together, TRACE-DDI establishes a unified and interpretable framework for biologically grounded DDI prediction and offers new directions for integrating actionable biological context into artificial intelligence-driven pharmacology.

## Highlights

• Pathway-anchored KG fusion: DRKG subgraphs are anchored at pathway nodes with a shortest-path constraint, so predicted DDIs can be traced through pathway mediators on the graph
• Centrality-guided interpretability: Shared pathways are ranked using multiple centrality measures to identify topologically prominent pathways as hypothesis-generating biological contexts
• Hybrid molecular encoder: A Transformer captures long-range SMILES dependencies, while a GAT encodes local chemical topology on SMILES-derived molecular graphs

## Introduction

Drug–drug interactions (DDIs) pose substantial clinical risks by causing adverse effects or reducing therapeutic efficacy when multiple drugs are coadministered [1]. With the increasing adoption of combination therapies [2], exhaustive experimental evaluation of all drug pairs is infeasible due to the associated cost and time demands [3,4]. As a result, computational approaches based on machine learning and deep learning have become essential for rapid, large-scale DDI prediction.

Recent studies span diverse strategies, yet notable limitations remain. Substructure–Substructure Interactions (SSI)-DDI (Nyamabo *et al.* [5]) identifies frequent molecular fragments in Simplified Molecular Input Line Entry System (SMILES) and models their pairwise interactions, but it lacks biological context (e.g., pathways or targets) and thus struggles to capture network-mediated effects. MUFFIN (Chen *et al.* [6]) fuses SMILES features, extracted via a one-dimensional convolutional neural network (1D-CNN), with knowledge-graph embeddings; however, 1D-CNN encoders treat SMILES as linear sequences and ignore graph topology, while simple element-wise fusion cannot model higher-order cross-modal interactions. Gated Message Passing Neural Network plus prediction module (GMPNN-CS; Nyamabo *et al.* [7]) applies a graph neural network with size-adaptive substructures to capture local molecular topology, but it does not integrate external knowledge graph (KG) information, resulting in incomplete global biological context. Caster (Huang *et al.* [8]) learns embeddings of chemical substructures extracted from SMILES, yet by treating molecules as sequences of substructures, it fails to preserve full graph connectivity and higher-order motifs.

More recent studies have explored stronger pairwise representation learning from molecular structure. Taco–DDI (Qiao *et al.* [9]) employs a graph transformer with dynamic coattention to model interactions directly from paired molecular graphs, improving DDI event prediction through layer-wise interaction-aware encoding. However, it remains primarily structure-centric and does not explicitly incorporate pathway-level or knowledge-graph-grounded biological context [9]. Similarly, Dual-Drug Visual Representation (DDVR–DDI; Xie *et al.* [10]) introduces a dual-drug visual representation that encodes drug pairs as concatenated molecular images and leverages self-supervised visual learning for DDI prediction. While innovative in its use of image-based structural cues, it also lacks explicit biological grounding and pathway-aware mechanistic interpretation.

Building on these studies, we are guided by 3 questions. First, how can explicit molecular graph topology be incorporated alongside SMILES sequences to capture both local atomic connectivity and nonlinear structural relationships often overlooked in sequential models? Second, how can knowledge-graph embeddings be effectively integrated with molecular representations so that predictive models gain both accuracy and biological interpretability? Finally, can a single end-to-end framework that jointly considers chemical structure and biological context outperform modular pipelines that treat these aspects in isolation?

We propose TRACE-DDI (TRAnsformer-GAT Context Encoder and pathway-anchored knowledge graphs for DDI prediction), a unified framework that combines molecular and biological representations within one architecture. The framework introduces a graph-aware encoding process that converts SMILES into both token sequences and adjacency matrices, preserving molecular topology such as rings, branches, and bond multiplicities. It then applies a hybrid encoder in which a Transformer captures long-range token dependencies and a Graph Attention Network (GAT) focuses on local atomic neighborhoods, yielding complementary molecular representations. In parallel, biological knowledge is incorporated by embedding subgraphs sampled from the Drug Repurposing Knowledge Graph (DRKG), thereby enriching molecular features with multirelational biological context. Unlike prior methods that emphasize only structural or visual pair representations, TRACE-DDI explicitly connects drug pairs through pathway-anchored substructures in the knowledge graph, enabling both improved predictive accuracy and more biologically grounded interpretation. This design improves predictive accuracy and, critically, supports interpretability through pathway visualization and centrality analysis in merged drug subgraphs.

### Key features

• Pathway-anchored KG integration (enabler of interpretability): We explicitly anchor DRKG subgraphs at pathway nodes and impose a shortest-path constraint between drug pairs.
  This design makes shared pathways emerge as candidate bridging contexts of predicted DDIs on the graph, providing a structured handle for hypothesis-generating interpretation.
• Centrality-based explanation (enabled by the pathway anchor): Because pathways occupy bridging positions in the merged subgraphs, we quantify their topological prominence using eigenvector, betweenness, degree, and closeness centralities, and rank shared pathways that are structurally central between the 2 drug neighborhoods.
• Hybrid molecular encoding: SMILES are processed by a Transformer (sequence semantics) and a GAT defined over the SMILES-derived molecular graph (local topology), yielding complementary drug-level embeddings.
• Unified multimodal fusion: Drug-level sequence, topology, and pathway-anchored KG context are fused in a single end-to-end classifier that supports both accurate prediction and pathway-level interpretability.

Together, these features form an end-to-end architecture that bridges molecular and knowledge-graph representations, advancing both the accuracy and interpretability of DDI prediction.

Beyond serving as a filtering mechanism, pathway anchoring introduces a biologically grounded structural prior into TRACE-DDI. Biological pathways represent functionally organized molecular processes and provide an intermediate abstraction layer between individual genes and higher-level phenotypes. By constraining subgraph extraction around pathway nodes, the framework induces a coherent mediator structure (Drug A–Pathway–Drug B), rather than relying on arbitrary graph proximity. This constraint reduces noise in large heterogeneous graphs, improves subgraph consistency, and enables biologically grounded interpretation through structured graph analysis.

The following sections present the model design in detail, describe the experimental setup, and evaluate its performance against existing baselines.

## Materials and Methods

**Fig. 1.**
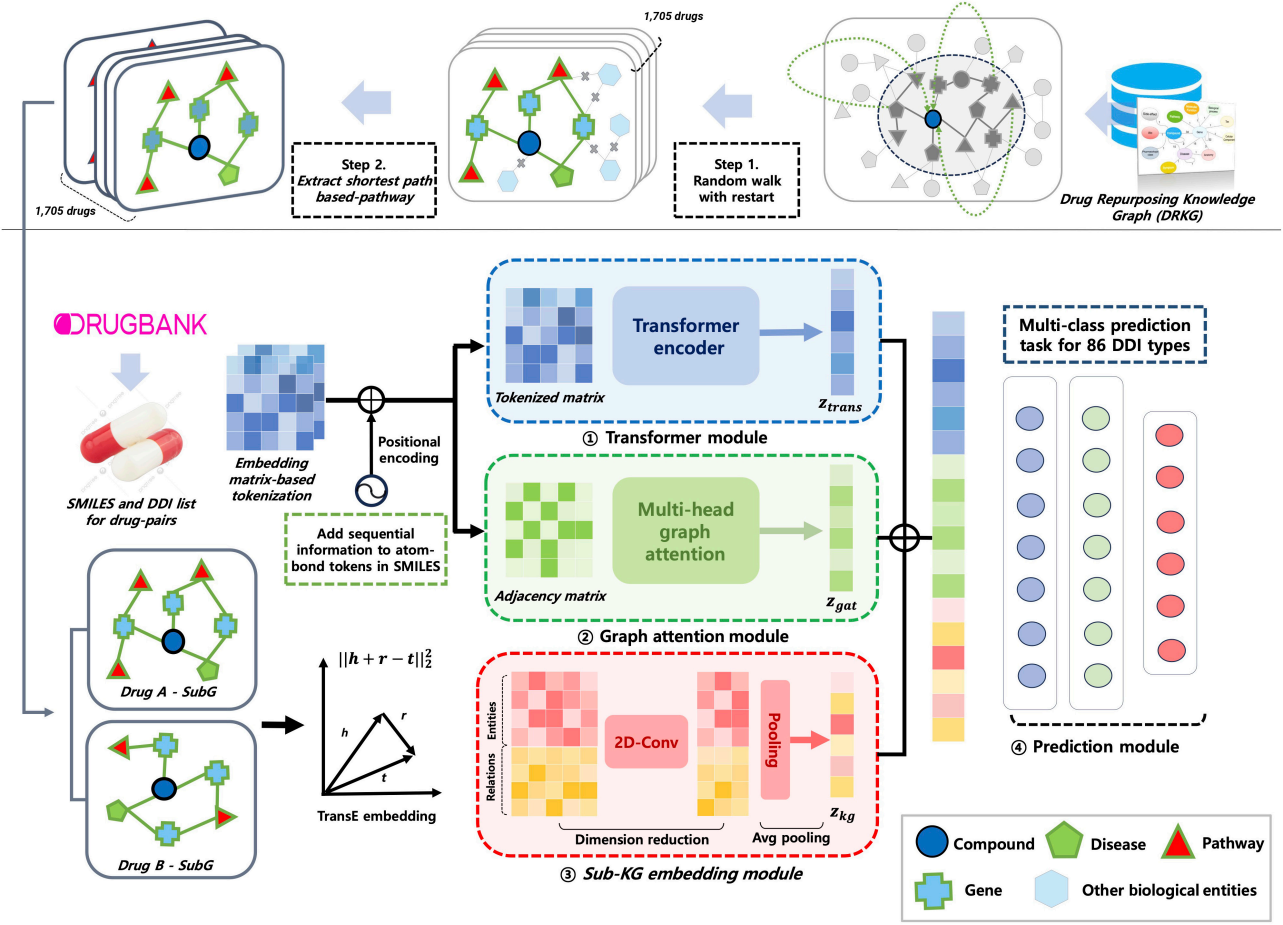
Overall workflow of TRACE-DDI, including SMILES tokenization, Transformer + GAT molecular encoding, and pathway-anchored KG fusion with shortest-path filtering for interpretable DDI prediction.

Figure 1 overviews the TRACE-DDI workflow with 5 stages: (a) preprocessing and filtering of DRKG to extract pathway-anchored, drug-centric subgraphs; (b) SMILES tokenization and Transformer-based sequence encoding; (c) graph construction from SMILES and GAT encoding of the molecular graph; (d) pathway-anchored DRKG subgraph embedding via Conv2D→GAP pooling (size-invariant); and (e) integration of all embeddings in a multiclass classifier for DDI prediction. The “Data preprocessing and filtering” to “Multiclass DDI classifier” sections detail each module, and the “Experimental settings” section covers experimental settings.

### Data preprocessing and filtering

We curate the DDI data and DRKG jointly so that sequence- and graph-level inputs are consistent. The DDI dataset used in this study is derived from DrugBank and follows the benchmark setting adopted in prior DDI prediction studies. The initial DDI file contains 1,706 drugs and 191,417 labeled pairwise interactions spanning 86 interaction types [3,11]. The complete list of the 86 interaction types is provided in Table S1. A summary of the DDI/SMILES filtering outcome is provided in Table 1.

**Table 1.** DDI/SMILES filtering summary

Item	Initial	Filtered
Drugs	1,706	1,692
DDIs	191,417	191,096

#### DRKG entity and edge filtering

Starting from the full DRKG [12], we retain biologically relevant entity families: compounds, pathways, genes, diseases, and side effects. An edge is kept only if both head and tail belong to these families. To analyze the impact of including side-effect nodes, we prepare 2 filtered variants used throughout the paper: Compound–Gene–Pathway–Disease–Side effect (CGPDS), which includes side effects, and Compound–Gene–Pathway–Disease (CGPD), which excludes them. The resulting node/edge counts for these variants are reported in Table 2.

**Table 2.** Summary of node/edge counts through each filtering step and random walk

Filtering/Step	#Nodes	#Edges
Initial graph	97,243	5,719,674
After filtering (CGPDS)	76,164	4,224,486
After filtering (CGPD)	70,463	4,085,542
Random walk (CGPDS, mean)	4,325.34	1,063,735.94
Random walk (CGPD, mean)	4,142.19	1,027,758.14
Final (4-hop, CGPDS, mean)	45.60	57.79
Final (4-hop, CGPD, mean)	50.55	65.45

#### Random walks and 4-hop subgraphs

To obtain drug-centric neighborhoods, we run random walks with step = 20,000, probability = 0.3, iterations = 10,000, and spread = 10^6^. A small subset of drugs appears as nodes but lacks incident edges after filtering; to avoid degenerate behavior during the walks, we attach a self-loop to each of them. For every drug, we then extract a subgraph by retaining nodes within 4 hops of pathway nodes encountered by the walks [13,14]. Summary statistics before and after this 4-hop extraction are shown in Table 2. These random-walk settings were chosen to provide sufficiently broad exploration of drug-centered neighborhoods in the large heterogeneous DRKG while maintaining stable pathway reachability across compounds. In a limited sensitivity check with representative parameter changes, low-budget sampling produced substantially more pathway-unreachable compounds, whereas increasing the sampling budget by either longer walks or more iterations led to a similar reduction in unreachable compounds. Additional details are provided in Section S4.

#### DDI/SMILES consistency filtering

We align the tabular DDI/SMILES data with the graph constraints above. Drugs that (a) still have no usable edges in the filtered DRKG after the walk preparation or (b) have no pathway reachable within a shortest path of at most 4 hops are removed from downstream modeling. In total, 14 drugs meet one of these exclusion criteria. After removal, the DDI file contains 191,096 interactions, and the SMILES list reduces from 1,706 to 1,692 drugs, as summarized in Table 1.

### SMILES tokenization and transformer encoding

Each drug is represented by its SMILES string [15,16], which we tokenize using a single, compiled regular expression with optional case sensitivity. We reserve 4 special tokens: <PAD>, <UNK>, <CLS>, and <SEP>. The tokenizer recognizes multicharacter atom symbols (e.g., Br and Cl), bracketed atom groups (e.g., [nH]), ring closures (digits), bond markers (-, =, #, and :), branch symbols ((, )), chirality indicators (e.g., @, \, and /), and generic alphanumeric tokens. All distinct tokens discovered in the corpus are added to the vocabulary; unseen tokens map to <UNK>.

For each SMILES, we prepend <CLS>, truncate or pad to a fixed length L (default L=128), and convert tokens to integer IDs. Sequences are embedded by a learned token-embedding matrix of dimension $d_{\mathrm{emb}}$, followed by a linear projection to the Transformer hidden size $d_{\text{model}}$. We apply a padding mask so that attention ignores <PAD> positions.

The projected sequences are then encoded by a stack of N Transformer encoder layers (batch-first) [17], each with $n_{\text{head}}$ self-attention heads and feed-forward dimension $d_{\mathrm{ff}}$. In TRACE-DDI, positional information is not used as a standard positional-encoding module whose primary purpose is to enhance Transformer sequence modeling. Instead, position-aware signals are introduced to preserve structurally distinguishable SMILES-derived token representations for the downstream token-to-graph and GAT-based encoding process. Accordingly, its role in our framework is tied primarily to maintaining an order-aware structural basis for graph construction and topology-aware molecular representation learning, while the Transformer branch captures sequence-level symbolic dependencies. This design separates sequence-level symbolic modeling from topology-aware graph reasoning. The encoder outputs a tensor of shape $\left(B,\;L,\;d_{\text{model}}\right)$. We use the <CLS> embedding as the sequence-level representation (the Transformer vector):

$$z_{\text{trans}}\in {\mathbb{R}}^{d_{\text{model}}}.$$ (1)

Although the Transformer branch is used to capture sequence-level symbolic dependencies in SMILES, the positional signal in TRACE-DDI is particularly important because it preserves order-aware structural distinctions among token representations before they are consumed by the downstream graph construction and GAT modules. Chemically meaningful topology is then modeled explicitly through adjacency-constrained graph encoding.

#### Module-specific note

The sequence encoder captures symbolic patterns and long-range dependencies in SMILES. Explicit molecular topology is modeled in the subsequent GAT module. The resulting sequence-level vector $z_{\text{trans}}$ is later concatenated with the GAT-based structural vector $z_{\mathrm{gat}}$ and the DRKG-derived vector $z_{\mathrm{kg}}$, enabling complementary signals to be fused without redundancy.

### Graph construction and GAT encoding over SMILES-derived molecular graphs

To make molecular geometry explicit ahead of graph reasoning, we reanalyze each tokenized SMILES to produce (a) a fixed-size adjacency matrix $A\in {\mathbb{R}}^{L\times L}$ aligned to the maximum token length *L*, and (b) position-aware node features for atom tokens that will seed the subsequent GAT. Tokens that correspond to atoms are treated as graph nodes; edges are created from bond markers (single, double, triple, and aromatic), ring-closure digits, and branch delimiters. Edge weights follow bond multiplicities, and self-loops can be added for numerical stability.

At the graph-construction stage, we introduce position-aware node features using a sinusoidal encoding with exponential decay. These positional signals are used to preserve order-aware token representations for the downstream graph-construction and graph-encoding process, rather than as a generic positional module introduced solely for sequence modeling in the Transformer encoder. For position *pos* and channel index *k* (with feature width *d*):

$$\begin{array}{c} \begin{array}{cc} \mathrm{PE}\left(\mathrm{pos},\;2k\right)\; & =\sin\;\left(\frac{\mathrm{pos}}{10000^{2k/d}}\right)\;\left(1+e^{-\mathrm{pos}/100}\right),\\ \mathrm{PE}\left(\mathrm{pos},\;2k+1\right)\; & =\cos\;\left(\frac{\mathrm{pos}}{10000^{2k/d}}\right)\;\left(1+e^{-\mathrm{pos}/100}\right). \end{array} \end{array}$$ (2)

Let $P\in {\mathbb{R}}^{L\times d}$ stack these encodings by row, and let $E\in {\mathbb{R}}^{L\times d}$ be the token embeddings at atom-token positions (nonatom rows are zeroed for graph construction). We form the position-aware node features

$$\tilde{H}\;=\;E\;+\;P,$$ (3)

which will be consumed by the GAT together with A.

To assess the contribution of this positional signal, we conducted an additional ablation experiment in which the position-aware token representations used for the downstream graph construction and GAT branch were removed while keeping the remaining TRACE-DDI architecture unchanged. This modification led to a substantial performance drop (Accuracy = 0.5159, weighted F1 = 0.4800, and macro F1 = 0.1774), indicating that positional information is critical for preserving structurally meaningful distinctions among SMILES-derived tokens in the token-to-graph encoding pipeline. Detailed results are provided in Section S9.

#### GAT encoding

Given $\left(A,\;\tilde{H}\right)$, we apply a *K*-head GAT to obtain a structure-aware representation. For head *k*,

$$\begin{array}{c} \alpha_{\mathrm{ij}}^{\left(k\right)}=\\ {\text{softmax}}_{j}\left(\text{LeakyReLU}\left(a_{k}^{⊤}\left[W_{k}h_{i}∥W_{k}h_{j}\right]\right)\right),\;\left(i,\;j\right)\in E \end{array}$$ (4)

and

$$h_{i}^{\prime }=\;{∥}_{k=1}^{K}\;\sigma \;\left(\sum_{j\in N\left(i\right)}\alpha_{\mathrm{ij}}^{\left(k\right)}\;W_{k}h_{j}\right).$$ (5)

We then apply readout (mean/max) with a residual MLP to produce $z_{\mathrm{gat}}\in {\mathbb{R}}^{Kd^{\prime }}$, which is later concatenated with the Transformer and KG vectors.

In contrast to sequence-based attention, this graph encoder aggregates information strictly along chemically valid bonds defined in the adjacency matrix.


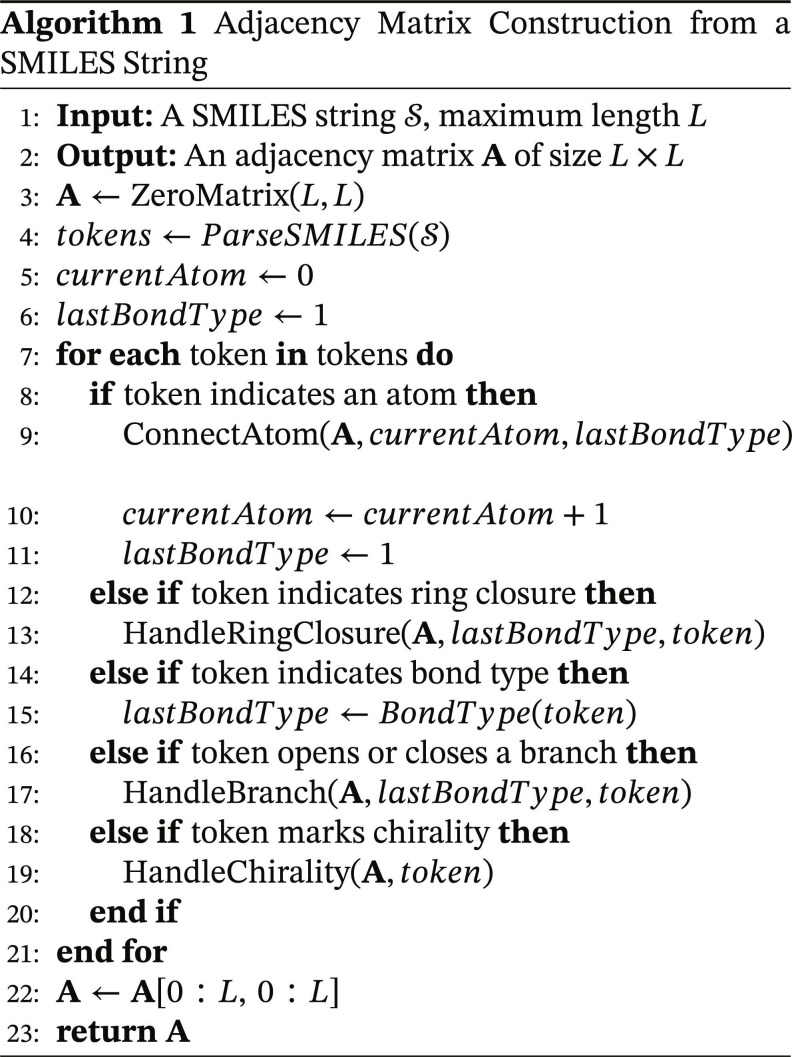


#### Module-specific note

Unlike the sequence-based Transformer, which operates over token positions in a linearized SMILES string, the GAT aggregates information strictly along bond-defined neighborhoods specified by the adjacency matrix A. Attention over token positions does not explicitly encode bond connectivity (e.g., branching or ring closures), whereas message passing over A reflects true atomic adjacency. Importantly, token proximity in SMILES does not necessarily correspond to graph proximity: atoms that are adjacent in the molecular graph can be far apart in the SMILES string due to branching notation, and ring-closure digits can connect atoms that are separated by many tokens. Therefore, adjacency-constrained message passing provides an explicit inductive bias for local chemical neighborhoods (bond types and multiplicities) that cannot be guaranteed by sequence-level self-attention alone. This design assigns complementary roles to the sequence and graph encoders, reducing redundancy by separating sequence-level semantics from explicit molecular topology.

To further validate the correctness of the SMILES-to-graph construction procedure, we conducted an additional experiment in which molecular graphs were generated using RDKit instead of the custom parser. All other components of the TRACE-DDI framework were kept unchanged. The RDKit-based model achieved strong predictive performance comparable to the main configuration, confirming that the overall framework does not depend on a specific graph-construction implementation. Detailed experimental results are reported in Section S7.

### External drug vectors (knowledge graph embedding)

Objective: We derive a compact, size-agnostic knowledge-graph vector for each drug by transforming its DRKG subgraph into a fixed-length embedding $z_{\mathrm{kg}}\in {\mathbb{R}}^{20}$. The subgraph is represented with pretrained TransE embeddings (400-D) for all entities and relations reachable by our preprocessing (random walks + 4-hop extraction).

TransE background: For each triple $\left(h,r,t\right)$, TransE assigns

$$f_{r}\left(h,\;t\right)=∥\;h+r-t\;{∥}_{2}^{2},$$ (6)

learned with a margin-based ranking loss over positive/corrupted triples [18]. We reuse the trained 400-D vectors for both entities and relations.

Subgraph matrix construction: For drug d, let $G_{d}$ be its filtered subgraph with entity set $E_{d}$ and relation set $R_{d}$. We stack the corresponding TransE vectors row-wise to form

$$M_{d}\in {\mathbb{R}}^{S\times 400},\;S=|E_{d}|+|R_{d}|.$$ (7)

This preserves the full, order-agnostic inventory of subgraph items without pre-aggregation (no sum/mean/principal component analysis [PCA] before projection).

Design rationale (Conv2D→GAP→FC): We treat the subgraph embedding matrix $M_{d}\in {\mathbb{R}}^{S\times 400}$ as a variable-sized set of entity and relation embeddings rather than as a spatial grid with semantic locality. Accordingly, the Conv2D layer is not intended to model graph topology or spatial adjacency in an image-like sense. Instead, it functions as a lightweight, learnable aggregation module that applies nonlinear feature transformation prior to global pooling.

Compared with fixed commutative reductions (e.g., sum or mean pooling) or linear compression methods (e.g., PCA), the Conv2D block enables learnable interactions across embedding dimensions before size-invariant summarization. Subsequent adaptive global average pooling removes dependence on subgraph size S and reduces sensitivity to row ordering at the global representation level. Thus, the Conv2D**→**GAP design should be understood as a parameter-efficient set-to-vector projection mechanism rather than a spatial reasoning model.

The ordering of rows in $M_{d}$ is deterministic but not assumed to encode biological hierarchy or spatial semantics. Spatial proximity in $M_{d}$ is not assumed to reflect semantic adjacency in the original knowledge graph; semantic structure is instead inherited from the pretrained TransE embedding space. To further assess the Conv-based KG aggregation design, we compared it with simpler alternatives including mean pooling, sum pooling, and PCA-based reduction under the same shared training configuration while keeping the remaining TRACE-DDI architecture unchanged. In this controlled setting, the Conv-based module remained highly competitive, showing performance very close to mean pooling while outperforming PCA-based reduction and sum pooling. These results indicate that the aggregation choice affects downstream performance and support the Conv block as a competitive learnable aggregation strategy for summarizing heterogeneous entity–relation subgraph embeddings, although simpler fixed reductions can also be effective. Detailed results are provided in Section S8.


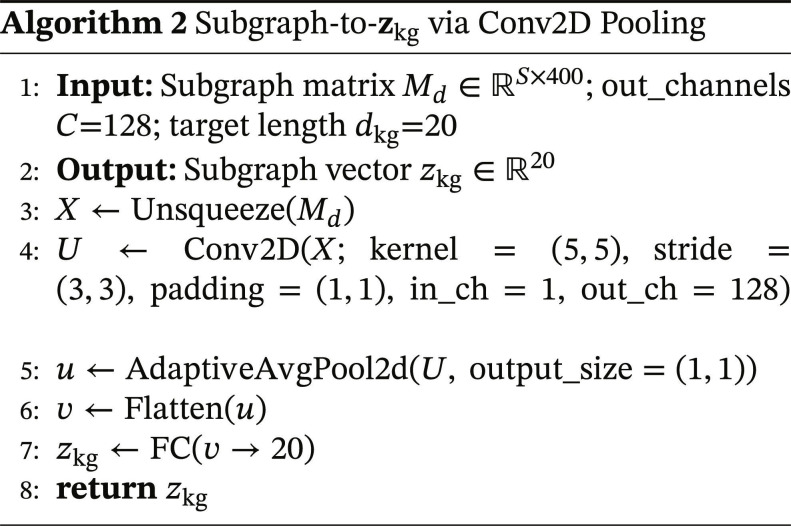


Implementation details (mirrors Algorithm 2). We reshape $M_{d}$ to $\left(1,\;1,\;S,\;400\right)$, apply Conv2D with kernel 5×5, stride 3×3, padding 1×1, out_channels C=128, then $\text{AdaptiveAvgPool}2d\left(1,1\right)$ to obtain a vector in ${\mathbb{R}}^{128}$. A fully connected layer produces $z_{\mathrm{kg}}\in {\mathbb{R}}^{20}$. This mapping is size-invariant (no padding/truncation needed) and integrates seamlessly with the downstream fusion/classifier.

The pathway-anchored DRKG subgraphs and their corresponding knowledge-graph embeddings are constructed globally from external biomedical resources and remain fixed across cross-validation folds. No DDI labels, validation data, or other fold-specific information are used during this preprocessing stage. During model training and evaluation, these KG-derived representations are treated as static input features and are not recomputed or fine-tuned on a per-fold basis.

#### Module-specific note

The vector $z_{\mathrm{kg}}$ summarizes the multirelational neighborhood of each drug and complements the sequence-level Transformer representation and the structure-aware GAT representation in our fusion-based DDI classifier.

### Multiclass DDI classifier

We fuse the 3 embeddings produced for each drug and perform multiclass classification on drug pairs. For a pair $\left(a,\;b\right)$, let $z_{\text{trans}}^{\left(a\right)}\in {\mathbb{R}}^{d_{\text{model}}}$, $z_{\mathrm{gat}}^{\left(a\right)}\in {\mathbb{R}}^{Kd^{\prime }}$, and $z_{\mathrm{kg}}^{\left(a\right)}\in {\mathbb{R}}^{d_{\mathrm{kg}}}$ denote the Transformer, GAT, and KG vectors for drug a; define the corresponding vectors for drug b analogously. Construct the joint feature by concatenation:

$$\begin{array}{c} x=\left[z_{\text{trans}}^{\left(a\right)}∥z_{\mathrm{gat}}^{\left(a\right)}∥z_{\mathrm{kg}}^{\left(a\right)}∥z_{\text{trans}}^{\left(b\right)}∥z_{\mathrm{gat}}^{\left(b\right)}∥z_{\mathrm{kg}}^{\left(b\right)}\right]\in {\mathbb{R}}^{D}, \end{array}$$ (8)

where $D=2\left(d_{\text{model}}+Kd^{\prime }+d_{\mathrm{kg}}\right)$.

The classifier is a multilayer fully connected network with batch normalization, LeakyReLU activations, and dropout. Denoting the network by $f_{\theta }:{\mathbb{R}}^{D}\to {\mathbb{R}}^{C}$ (with C interaction types), class probabilities are

$$p=\text{softmax}\;\left(f_{\theta }\left(x\right)\right),$$ (9)

and training minimizes cross-entropy using Adam [19].

#### Module-specific note

Concatenating the Transformer, GAT, and KG vectors from both drugs aggregates complementary semantic, structural, and biological-context signals into a single representation.

### Experimental settings

We evaluate our framework under the following design:

• Stratified 5-fold cross-validation: The dataset is split into 5 folds via stratified sampling, preserving class distributions.
• Early stopping: Training halts if validation accuracy (val_acc) does not improve for 10 consecutive epochs, and the checkpoint corresponding to the best validation epoch is retained for evaluation. This procedure serves as an implicit regularization mechanism to limit late-stage overfitting in the multimodal architecture.
• Hyperparameter tuning: Hyperparameters were optimized using Optuna-based automated search over predefined ranges for learning rate, hidden dimensions, number of attention heads, and dropout rate. The objective function was defined on validation performance within stratified cross-validation, and early stopping was applied to prevent overfitting.
• Logging and checkpointing: Training logs are stored in CSV format, and model checkpoints are saved at the best epoch per fold.
• Evaluation metrics: We report accuracy, F1-score, precision, and recall; a per-class report provides detailed breakdowns.

We implement our approach in PyTorch Lightning with support for multiple graphics processing units (GPUs) [20]. The modular design of the training loop—encompassing data loading, model instantiation, training, and evaluation—ensures reproducibility and facilitates future expansion. All experiments were conducted with fixed random seeds for data splitting, model initialization, and sampling procedures. The public repository includes the preprocessing pipeline and training scripts used to reproduce the reported results. Experiments were conducted on a Linux server equipped with dual Intel Xeon Gold 5120 CPUs, 376 GB RAM, and a single NVIDIA GeForce RTX 4090 GPU (24 GB). Under the best-performing setting, model training required approximately 2.5 GB of GPU memory and took approximately 30 h on a single GPU. In contrast, the random-walk-based subgraph construction stage was substantially more computationally demanding, requiring approximately 120 to 136 h under the hyperparameter configuration used in this study.

Complete baseline configurations, architecture summaries, and detailed hyperparameter settings for all compared models are provided in Section S2.

## Results and Discussion

Workflow summary: Figure 1 provides a high-level overview of the TRACE-DDI pipeline, covering each stage from SMILES-based graph construction to knowledge-graph fusion and final classification. After converting SMILES to both sequence and adjacency representations, we incorporate relational data via random-walk sampling from DRKG. This end-to-end approach systematically captures both local molecular detail and broader biological context.

### Performance comparison with baseline models

Table 3 presents a quantitative comparison between TRACE-DDI and several strong baselines—Caster, GMPNN-CS, MUFFIN, and SSI-DDI—trained and evaluated on the same dataset split for a fair assessment. TRACE-DDI achieves the highest accuracy (0.9746) and F1-score (0.9741), surpassing each baseline by a notable margin, underscoring the efficacy of our integrated approach.

**Table 3.** Comparison of baseline methods and our TRACE-DDI model. F1, Precision, and Recall for all baseline models were obtained by reimplementing and evaluating them on the same dataset splits as TRACE-DDI for a fair comparison.

Method	ACC	F1	Precision	Recall
Caster	0.8296	0.8300	0.8320	0.8280
GMPNN-CS	0.9516	0.9510	0.9520	0.9500
MUFFIN	0.9457	0.9450	0.9470	0.9440
SSI-DDI	0.9015	0.9000	0.9020	0.8990
TRACE-DDI	0.9746	0.9741	0.9747	0.9752

ACC, classification accuracy

Given the pronounced long-tail distribution across the 86 interaction types, we additionally report macro-averaged metrics and per-class performance. The observed gap between weighted and macro-averaged scores reflects the inherent difficulty of minority interaction types rather than performance inflation driven by dominant classes.

Because the primary 86-class benchmark uses pair-level stratified cross-validation, it should be interpreted as a transductive evaluation setting rather than a strict unseen-drug setting. Detailed fold-wise drug overlap statistics and fold-wise overall performance results are provided in Section S5. Accordingly, the very high performance observed in this benchmark should be interpreted as strong transductive performance on new drug pairs formed largely from previously observed drugs, rather than as evidence of strong inductive generalization to completely unseen compounds.

This protocol was adopted because the 86-class benchmark exhibits pronounced long-tail imbalance, and strict drug-level splitting can remove rare interaction types from either training or evaluation folds, making multiclass learning unstable. We therefore retain pair-level stratified evaluation as the primary class-support-preserving benchmark, while reporting a complementary strict unseen-drug experiment in Section S6 to assess inductive generalization.

### Ablation study

To explicitly assess the relative contribution of each component, we conducted an ablation study summarized in Table 4.

**Table 4.** Ablation results on CGPDS and CGPD. Each cell shows average Accuracy/F1/Precision/Recall over 5 folds.

Method	CGPDS	CGPD
Transformer only	0.9498/0.8466/0.8493/0.8498	0.9377/0.8472/0.8411/0.8529
GAT only	0.9470/0.9465/0.9472/0.9470	0.9525/0.9524/0.9525/0.9525
Transformer + GAT	0.9549/0.9527/0.9411/0.9426	0.9538/0.9536/0.9538/0.9538
KG only (rw)	0.8876/0.8872/0.8891/0.8876	0.8871/0.8868/0.8889/0.8871
KG only (rw + shortest path)	0.8870/0.8866/0.8887/0.8870	0.8877/0.8872/0.8891/0.8877
Transformer + KG (rw)	0.9608/0.9607/0.9610/0.9618	0.9674/0.9673/0.9676/0.9674
Transformer + KG (rw + shortest path)	0.9674/0.9674/0.9676/0.9674	0.9672/0.9672/0.9674/0.9672
TRACE-DDI (ours)	0.9746/0.9741/0.9747/0.9752	0.9751/0.9734/0.9761/0.9772

The Transformer-only model serves as a molecular baseline, capturing sequence-level semantic information from SMILES. While it achieves strong performance, its results indicate that sequence modeling alone is insufficient to fully explain DDIs.

GAT-only represents a topology-driven molecular encoder that relies exclusively on bond-constrained message passing over the SMILES-derived molecular graph. Although it ignores sequence-level symbolic patterns, it achieves competitive performance, indicating that explicit molecular topology alone provides strong predictive signals for DDI classification.

The Transformer + GAT variant further supports the complementarity between sequence-level and topology-aware molecular encoding. Across both CGPDS and CGPD settings, this hybrid molecular variant outperformed the single-modality molecular encoders, indicating that symbolic SMILES dependencies and bond-constrained graph structure provide mutually supportive information even before the integration of KG-based biological context. However, its performance remained below that of the full TRACE-DDI model, showing that pathway-anchored biological context provides additional gains beyond molecular representation alone.

The KG-only variants (rw and rw + shortest path) demonstrate that biological relational context contains informative signals. However, their performance remains below that of the Transformer-only model, suggesting that knowledge-graph context alone cannot adequately capture interaction-specific chemical patterns.

When combining modalities, the Transformer + KG configurations yield a substantial improvement over either component alone. This performance gain highlights the complementary nature of molecular structure information and pathway-anchored biological context. In particular, the addition of shortest-path constraints further stabilizes and refines relational representations.

Finally, the complete TRACE-DDI model achieves the highest performance across both CGPDS and CGPD datasets. The additional improvement over Transformer + KG reflects the contribution of topology-aware molecular encoding via the GAT module. By introducing bond-constrained message passing, the GAT refines molecular representations beyond linear sequence modeling. Although this improvement is incremental relative to the multimodal fusion step, it is consistent across datasets, indicating that explicit molecular topology provides meaningful refinement.

Overall, the ablation results demonstrate that (a) molecular sequence information provides a strong foundation, (b) combining sequence-level and topology-aware molecular encoders yields additional gains, (c) pathway-anchored knowledge-graph context contributes substantial complementary improvement, and (D) the full multimodal integration achieves the best overall performance.

Additional implementation details and supporting statistics for the ablation experiments are provided in Section S2.

### Case studies

To minimize narrative bias, representative drug pairs were selected according to predefined quantitative criteria rather than retrospective biological interpretation. Predicted interacting pairs were not selected heuristically. Instead, for each anchor drug and interaction label, shared pathway candidates were first filtered by minimum coverage across interacting partner drugs and then ranked using a weighted separation score derived from centrality differences between interacting and noninteracting partner groups, with additional emphasis on interacting-group eigenvector prominence and pathway coverage. Candidate signals were subsequently screened using Mann–Whitney *U* statistics, Benjamini–Hochberg false discovery rate correction, effect-direction consistency, and effect-size thresholds across multiple centrality measures. Accordingly, the presented case studies were selected from a predefined quantitative screening pipeline rather than by retrospective narrative choice, and should be interpreted as hypothesis-generating examples rather than confirmatory evidence. A concise algorithm summary and pseudo-code for this case-study selection pipeline are provided in Section S3.

**Fig. 2.**
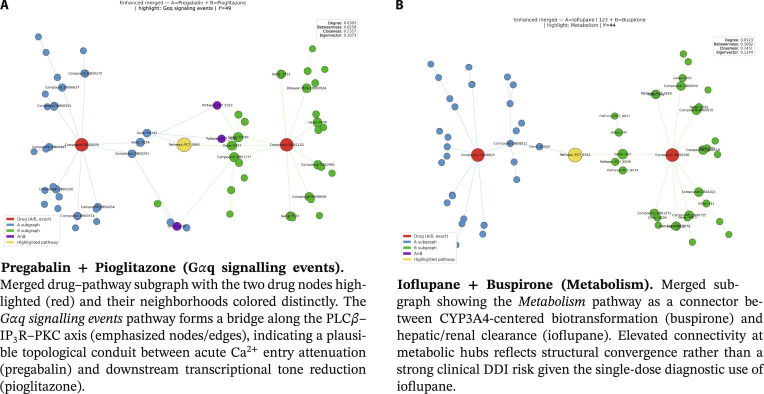
Merged subgraphs for 2 representative drug pairs. Shared pathway nodes lie at the interface of drug-specific neighborhoods and provide pathway-anchored context for interpretability. (A) Pregabalin + pioglitazone (G𝛼q signaling events). Merged drug–pathway subgraph with the two drug nodes highlighted (red) and their neighborhoods colored distinctly. The G𝛼q signaling events pathway forms a bridge along the PLC𝛽–IP3R–PKC axis (emphasized nodes/edges), indicating a plausible topological conduit between acute Ca^2+^ entry attenuation (pregabalin) and downstream transcriptional tone reduction (pioglitazone). (B) Ioflupane + buspirone (metabolism). Merged subgraph showing the Metabolism pathway as a connector between CYP3A4-centered biotransformation (buspirone) and hepatic/renal clearance (ioflupane). Elevated connectivity at metabolic hubs reflects structural convergence rather than a strong clinical DDI risk given the single-dose diagnostic use of ioflupane.

**Fig. 3.**
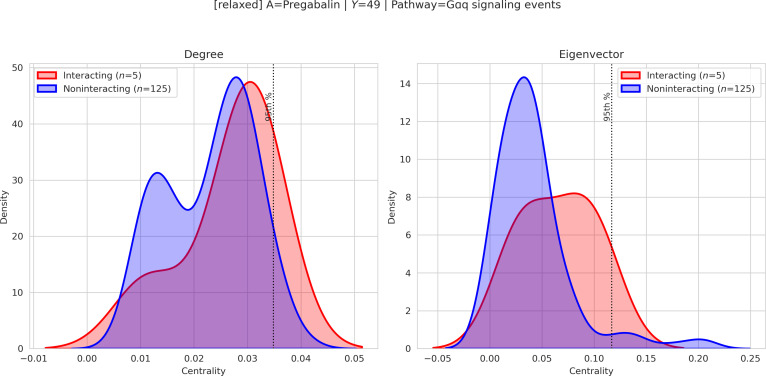
Kernel density estimation (KDE) of pathway centralities for the Pregabalin–Gαq signaling events pair. The *x*-axis represents the centrality score (degree or eigenvector) of the shared pathway within merged drug-centered subgraphs, and the *y*-axis denotes the estimated density. Red curves correspond to interacting drug pairs, while blue curves correspond to noninteracting pairs. The vertical dashed line marks the 95th percentile of the centrality distribution, and rug ticks indicate individual high-scoring observations. A relative right shift of the interacting distribution suggests that this pathway tends to occupy more central topological positions in predicted DDIs compared with noninteracting pairs.

**Fig. 4.**
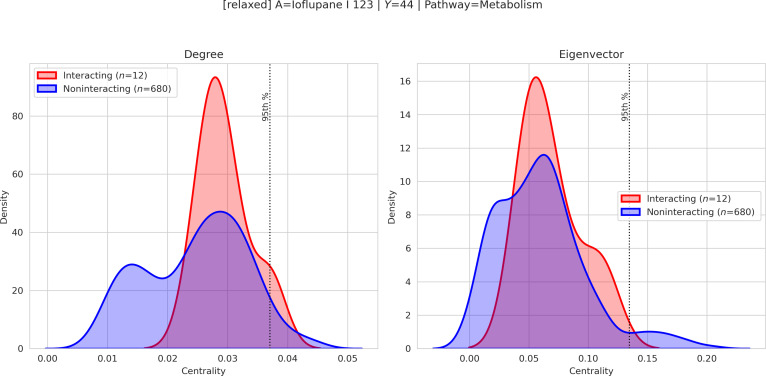
Kernel density estimation (KDE) of pathway centralities for the Ioflupane I-123–Metabolism pair. Centrality scores (degree and eigenvector) of the shared metabolic pathway are shown along the *x*-axis, with density estimates on the *y*-axis. Red curves indicate interacting drug pairs, and blue curves indicate noninteracting pairs. The dashed vertical line denotes the 95th percentile threshold, and rug marks represent individual high-centrality instances. The observed distributional separation reflects a higher relative topological prominence of the metabolism pathway in interacting predictions, without implying direct causal mechanisms.

The following examples therefore illustrate instances of a broader quantitative pattern observed in the dataset, rather than isolated anecdotal findings. First, we visualize merged drug–pathway subgraphs to identify shared pathway nodes that topologically connect the 2 drugs (Fig. 2). Second, leveraging the pathway-anchored design, we compute centrality scores within the merged subgraphs and compare their distributions between interacting and noninteracting pairs (Figs. 3 and 4). The resulting topological patterns are interpreted in conjunction with prior biological evidence and are explicitly framed as hypothesis-generating observations rather than causal claims.

#### Case study 1: Pregabalin + pioglitazone (G𝞪q signaling events)

In the merged subgraph, the Gαq signaling events pathway occupies a prominent topological position, showing elevated betweenness and eigenvector centralities along the PLCβ–IP_3_R–PKC axis. The KDE distribution exhibits a right shift for interacting pairs, indicating that this pathway is preferentially highlighted in predicted DDIs.

From a biological perspective, pregabalin reduces presynaptic Ca^2+^ influx via binding to the $\alpha_{2}\delta$ subunit of voltage-gated calcium channels, thereby modulating excitatory neurotransmission [21,22]. Pioglitazone, a PPARγ agonist, regulates inflammatory transcriptional programs and attenuates NF-κB-related downstream signaling [23,24]. The identified Gαq–PLC–IP_3_–PKC signaling cascade provides a biologically plausible interface through which calcium-dependent signaling and transcriptional modulation may converge.

We emphasize that centrality does not imply causality; rather, the model highlights Gαq-related signaling as a candidate mediating context consistent with prior mechanistic reports. This interpretation should therefore be regarded as a hypothesis-generating insight supported by both graph topology and published biological evidence.

#### Case study 2: Ioflupane + buspirone (metabolism)

In the merged graph, the Metabolism pathway emerges as a shared topological connector between the 2 drugs, with elevated degree and eigenvector centralities relative to noninteracting pairs. The KDE analysis similarly indicates preferential centrality enrichment in interacting predictions.

Biologically, buspirone undergoes extensive hepatic metabolism, primarily via CYP3A4-mediated biotransformation [25,26]. Ioflupane I-123, a diagnostic radiopharmaceutical, is cleared through hepatic and renal processes, with established pharmacokinetic safety profiles [27]. The shared metabolic context identified by TRACE-DDI reflects convergence on common clearance and biotransformation pathways.

However, given the single-dose diagnostic use of ioflupane, the clinical likelihood of substantial pharmacokinetic DDIs remains limited. Accordingly, the centrality-based signal should be interpreted as structural convergence within metabolic networks rather than definitive evidence of clinically meaningful interaction.

### Visualization of shared pathways in drug subgraphs

To investigate mechanistic overlap between drugs, we constructed subgraphs centered on each compound and extracted their shared pathways. Specifically, we merged 2 drug-centered subgraphs and highlighted common pathway nodes that may mediate pharmacological interactions [28,29]. In the merged graphs, drug-specific neighborhoods are colored distinctly (e.g., blue for Drug A and green for Drug B), while the drug nodes are highlighted in red. Shared pathways at the interface are emphasized for their bridging roles.

This visualization reveals whether 2 drugs are topologically connected through biological processes. Only when a pathway node is shared (i.e., Drug A–Pathway X–Drug B) do the subgraphs form an actual connection, providing a biologically interpretable context for potential DDIs. This forms the basis for subsequent quantitative analysis, prioritizing pathways that may mechanistically explain DDIs.

### Centrality score analysis

To quantify the structural importance of shared pathway nodes within merged drug-centered subgraphs, we computed 4 complementary centrality measures: degree, betweenness, closeness, and eigenvector centrality [30–32]. These metrics capture distinct aspects of network prominence: (a) degree reflects local connectivity within the subgraph, (b) betweenness quantifies the extent to which a pathway lies on shortest communication routes between drug-specific neighborhoods, (c) closeness measures how efficiently a pathway can reach other nodes in the merged structure, and (d) eigenvector centrality highlights influence based on connections to other highly connected nodes. In TRACE-DDI, pathway anchoring is intrinsic to the model architecture because it is incorporated during subgraph construction and knowledge-graph representation learning. By contrast, centrality analysis is applied post hoc after prediction as an interpretability layer and does not affect model optimization or parameter updates. Therefore, the reported interpretability results should be understood as post hoc, hypothesis-generating analyses of pathway-level graph context rather than as direct explanations of the model's internal decision process or as evidence of biological causation.

Because TRACE-DDI constructs pathway-anchored subgraphs that induce mediator structures of the form Drug A–Pathway–Drug B, centrality scores provide a quantitative means to evaluate whether a shared pathway occupies an influential bridging position between the 2 drugs. We therefore compared the distributions of pathway centralities between interacting and noninteracting drug pairs. Candidate shared pathways were first required to satisfy a minimum coverage criterion across interacting partner drugs. Distributional separation was then summarized using centrality-specific mean and median differences, and representative pathway candidates were ranked using predefined weights (REP_W = {betweenness: 0.40, eigenvector: 0.30, degree: 0.20, closeness: 0.10}).

Although all 4 centralities were computed and incorporated into the analysis, we visualize only degree and eigenvector centralities—those that exhibited the clearest separation between interacting and noninteracting pairs in our dataset (Figs. 3 and 4). The observed right shift of centrality distributions for interacting pairs indicates that shared pathways in predicted DDIs tend to occupy more topologically influential positions within merged subgraphs.

Thus, centrality analysis serves as a structured, graph-theoretic criterion to prioritize biologically plausible mediating pathways. Rather than implying causality, these measures support mechanism-oriented interpretation by identifying pathway nodes that structurally bridge drug-specific neighborhoods in predicted interactions.

To mitigate the risk that highly connected global hub pathways dominate the analysis irrespective of specific interactions, centrality scores were computed within drug-pair-specific merged subgraphs rather than on the global knowledge graph. Moreover, pathway centrality distributions were explicitly compared between interacting and noninteracting drug pairs, providing an internal control for evaluating interaction-specific structural prominence. Thus, the reported separation reflects relative distributional shifts conditioned on interaction labels, rather than absolute global pathway popularity.

Because pathway centralities are derived from overlapping drug-pair-specific subgraphs, the resulting samples are not strictly independent; therefore, the KDE plots are interpreted as descriptive visual summaries rather than stand-alone inferential tests. Formal statistical screening was instead applied at the candidate-selection stage using Mann–Whitney *U* statistics, Benjamini–Hoch-berg correction, and effect-size criteria, while the KDE figures are used to visualize the resulting separation patterns. Additional examples of anchor–pathway visualizations across multiple interaction labels are provided in Section S3.

To quantitatively evaluate the separation between interacting and noninteracting drug pairs, we computed centrality-specific statistical summaries for each candidate pathway, including the Mann–Whitney *U* test, Cliff's delta effect size, and centrality-wise mean and median differences. False discovery rate correction was applied using the Benjamini–Hochberg procedure across tested candidates. In our screening procedure, pathway signals were prioritized only when they showed favorable statistical evidence together with consistent positive effect direction across multiple centrality measures. Detailed numerical summaries and additional examples are provided in Section S3.

### Discussion and future directions

TRACE-DDI demonstrates consistent improvements in DDI prediction, supported by quantitative metrics and qualitative visualizations [1,33,34]. The ablation shows that incorporating random-walk sampling, shortest-path constraints, and external knowledge-graph embeddings yields substantial gains. Pathway-level visualizations reveal that high-centrality nodes frequently correspond to biologically plausible contexts, reinforcing interpretability while remaining hypothesis-generating in nature.

Key contributions:

• Unified integration of 3 modalities: We jointly model (a) the SMILES sequence, (b) the molecular graph topology of each drug, and (c) the pathway-anchored knowledge-graph context within a single end-to-end framework, effectively capturing both fine-grained chemical structure and global biological context.
• Enhanced predictive accuracy via cross-modal graph reasoning: By combining transformer-based encoding with deep graph reasoning across heterogeneous modalities, our model produces robust and biologically interpretable predictions. It consistently outperforms state-of-the-art baselines across all major evaluation metrics.
• Centrality-based visualization: TRACE-DDI prioritizes topologically prominent shared pathways through graph-theoretic centrality analysis, providing interpretable structural insights into predicted drug interactions without asserting causal mediation.

Applications and broader impact: The framework is readily extensible to drug repurposing, polypharmacy risk modeling, and protein–protein interaction discovery. Its modular design allows integration with additional modalities, including transcriptomic or proteomic networks and real-world electronic health records.

To further evaluate generalization to unseen compounds, we conducted an additional strict unseen-drug experiment under a binary DDI prediction setting. In this protocol, the complete drug set was partitioned into disjoint seen and holdout subsets, the model was trained only on seen–seen pairs, and evaluation was performed exclusively on holdout–holdout pairs, yielding zero overlap between training and test drugs by construction (holdout drugs: 338; shared drugs: 0). The same TRACE-DDI architecture and training configuration used for the original multiclass task were reused in this experiment except for replacing the final classification layer with a binary output layer, so the reported results should be interpreted as a conservative estimate of inductive generalization. Under this strict setting, TRACE-DDI achieved an accuracy of 0.5990, a receiver operating characteristic area under the curve (ROC-AUC) of 0.6541, a precision–recall area under the curve (PR-AUC) of 0.6725, a macro-F1 of 0.5828, and a weighted-F1 of 0.5803, indicating that the model retains meaningful predictive ability even when both drugs in the test pair are previously unseen during training. However, this performance remains substantially lower than that of the primary pair-level benchmark, indicating that generalization to completely unseen drugs is still limited relative to the transductive setting. Detailed experimental configuration, dataset statistics, and class-wise results for this strict unseen-drug evaluation are provided in Section S6.

Future work: We will explore advanced graph neural architectures (e.g., graph transformers such as Graphormer) for refined multimodal integration, while preserving the representational separation between sequence-level symbolic modeling and explicit bond-constrained topology encoding that motivates the current Transformer–GAT design. We will also scale to larger heterogeneous datasets and expand interpretability features (attention maps, node-level attribution, and causal analyses) to deepen mechanistic understanding. In addition, because TRACE-DDI combines multiple modules beyond a single stand-alone GNN, model-intrinsic attribution methods such as GNNExplainer or gradient-based node/feature attribution are not straightforwardly applicable in the current framework. Developing module-aware attribution strategies that more directly connect model predictions to contributing features and graph components therefore remains an important direction for future work. Although we compare interacting and noninteracting pairs as an internal control in the present study, incorporating randomized structural negative controls (e.g., degree-preserving rewiring or nonpathway-anchored subgraph construction) would further disentangle interaction-specific structure from generic graph topology and constitutes an important direction for future work.

## Conclusion

We presented TRACE-DDI, a unified framework that bridges molecular graph representations and knowledge-graph embeddings to predict DDIs. By coupling Transformer-based feature extraction with graph attention and random-walk-based sampling in a knowledge graph, the method captures both local chemical structure and global biological context. Extensive experiments, including cross-validation and ablations, confirm superior predictive accuracy relative to strong baselines. In addition, the pathway-anchored design provides a structured mechanism for interpretation: merged drug subgraphs expose shared pathway mediators, and pair-specific centrality analyses prioritize topologically prominent pathways as hypothesis-generating biological contexts rather than causal explanations. Visualizations of shared pathways and centrality analyses offer biologically meaningful explanations for model outputs, highlighting key metabolic and signaling routes in DDI phenomena. Nevertheless, we emphasize that these explanations are descriptive and depend on the constructed subgraphs; incorporating randomized structural negative controls (e.g., degree-preserving rewiring) and evaluating drug-level splits would further strengthen claims about interaction-specific biological signal and generalization. This framework lays groundwork for further innovations in multimodal data fusion, interpretability, and scalability [1,33].

## Data Availability

The code for the entire workflow used in this research is available at https://github.com/BML-CBNU/TRACE-DDI.
