# Supplementary material for: TRACE-DDI: A Hybrid Framework of Transformer–GAT Context Encoder and Pathway-Anchored Knowledge Graphs for DDI Prediction
Source: Comput Struct Biotechnol J. 2026 May 15;35(1):0057. doi: 10.34133/csbj.0057 (PMC13176605; doi:10.34133/csbj.0057)
Supplement: Supplementary 1 — Supplementary Text Tables S1 to S10 [file csbj.0057.f1.pdf]

Supplementary material

1. Drug–Drug Interaction Types

Table S1. Interaction types of the dataset.

Index	Description
1	Drug1 may increase the photosensitizing activities of Drug2
2	Drug1 may increase the anticholinergic activities of Drug2
3	The bioavailability of Drug2 can be decreased when combined with Drug1
4	The metabolism of Drug2 can be increased when combined with Drug1
5	Drug1 may decrease the vasoconstricting activities of Drug2
6	Drug1 may increase the anticoagulant activities of Drug2
7	Drug1 may increase the ototoxic activities of Drug2
8	The therapeutic efficacy of Drug2 can be increased when used in combination with Drug1
9	Drug1 may increase the hypoglycemic activities of Drug2
10	Drug1 may increase the antihypertensive activities of Drug2
11	The serum concentration of the active metabolites of Drug2 can be reduced when Drug2 is used in combination with Drug1
12	Drug1 may decrease the anticoagulant activities of Drug2
13	The absorption of Drug2 can be decreased when combined with Drug1
14	Drug1 may decrease the bronchodilatory activities of Drug2
15	Drug1 may increase the cardiotoxic activities of Drug2
16	Drug1 may increase the central nervous system depressant activities of Drug2
17	Drug1 may decrease the neuromuscular blocking activities of Drug2
18	Drug1 can cause an increase in the absorption of Drug2, resulting in an increased serum concentration
19	Drug1 may increase the vasoconstricting activities of Drug2
20	Drug1 may increase the QTc-prolonging activities of Drug2
21	Drug1 may increase the neuromuscular blocking activities of Drug2
22	Drug1 may increase the adverse neuromuscular activities of Drug2
23	Drug1 may increase the stimulatory activities of Drug2
24	Drug1 may increase the hypocalcemic activities of Drug2

25	Drug1 may increase the atrioventricular blocking activities of Drug2
26	Drug1 may decrease the antiplatelet activities of Drug2
27	Drug1 may increase the neuroexcitatory activities of Drug2
28	Drug1 may increase the dermatologic adverse activities of Drug2
29	Drug1 may decrease the diuretic activities of Drug2
30	Drug1 may increase the orthostatic hypotensive activities of Drug2
31	The risk or severity of hypertension can be increased when Drug2 is combined with Drug1
32	Drug1 may increase the sedative activities of Drug2
33	The risk or severity of QTc prolongation can be increased when Drug1 is combined with Drug2
34	Drug1 may increase the immunosuppressive activities of Drug2
35	Drug1 may increase the neurotoxic activities of Drug2
36	Drug1 may increase the antipsychotic activities of Drug2
37	Drug1 may decrease the antihypertensive activities of Drug2
38	Drug1 may increase the vasodilatory activities of Drug2
39	Drug1 may increase the constipating activities of Drug2
40	Drug1 may increase the respiratory depressant activities of Drug2
41	Drug1 may increase the hypotensive and central nervous system depressant (CNS depressant) activities of Drug2
42	The risk or severity of hyperkalemia can be increased when Drug1 is combined with Drug2
43	The protein binding of Drug2 can be decreased when combined with Drug1
44	Drug1 may increase the central neurotoxic activities of Drug2
45	Drug1 may decrease effectiveness of Drug2 as a diagnostic agent
46	Drug1 may increase the bronchoconstrictory activities of Drug2
47	The metabolism of Drug2 can be decreased when combined with Drug1
48	Drug1 may increase the myopathic rhabdomyolysis activities of Drug2
49	The risk or severity of adverse effects can be increased when Drug1 is combined with Drug2
50	The risk or severity of heart failure can be increased when Drug2 is combined with Drug1
51	Drug1 may increase the hypercalcemic activities of Drug2
52	Drug1 may decrease the analgesic activities of Drug2
53	Drug1 may increase the antiplatelet activities of Drug2

54	Drug1 may increase the bradycardic activities of Drug2
55	Drug1 may increase the hyponatremic activities of Drug2
56	The risk or severity of hypotension can be increased when Drug1 is combined with Drug2
57	Drug1 may increase the nephrotoxic activities of Drug2
58	Drug1 may decrease the cardiotoxic activities of Drug2
59	Drug1 may increase the ulcerogenic activities of Drug2
60	Drug1 may increase the hypotensive activities of Drug2
61	Drug1 may decrease the stimulatory activities of Drug2
62	The bioavailability of Drug2 can be increased when combined with Drug1
63	Drug1 may increase the myelosuppressive activities of Drug2
64	Drug1 may increase the serotonergic activities of Drug2
65	Drug1 may increase the excretion rate of Drug2 which could result in a lower serum level
66	The risk or severity of bleeding can be increased when Drug1 is combined with Drug2
67	Drug1 can cause a decrease in the absorption of Drug2 resulting in a reduced serum concentration
68	Drug1 may increase the hyperkalemic activities of Drug2
69	Drug1 may increase the analgesic activities of Drug2
70	The therapeutic efficacy of Drug2 can be decreased when used in combination with Drug1
71	Drug1 may increase the hypertensive activities of Drug2
72	Drug1 may decrease the excretion rate of Drug2 which could result in a higher serum level
73	The serum concentration of Drug2 can be increased when it is combined with Drug1
74	Drug1 may increase the fluid retaining activities of Drug2
75	The serum concentration of Drug2 can be decreased when it is combined with Drug1
76	Drug1 may decrease the sedative activities of Drug2
77	The serum concentration of the active metabolites of Drug2 can be increased when Drug2 is used in combination with Drug1
78	Drug1 may increase the hyperglycemic activities of Drug2
79	Drug1 may increase the central nervous system depressant (CNS depressant) and hypertensive activities of Drug2
80	Drug1 may increase the hepatotoxic activities of Drug2
81	Drug1 may increase the thrombogenic activities of Drug2
82	Drug1 may increase the arrhythmogenic activities of Drug2

83	Drug1 may increase the hypokalemic activities of Drug2
84	Drug1 may increase the vasopressor activities of Drug2
85	Drug1 may increase the tachycardic activities of Drug2
86	The risk of a hypersensitivity reaction to Drug2 is increased when it is combined with Drug1

2. Baseline Model Architectures and Hyperparameters

2.1 Concise model architecture summaries

TRACE-DDI (ours) — Hybrid sequence, topology, and biology.

The model converts SMILES (Simplified Molecular Input Line Entry System) into token sequences and an adjacency matrix. A Transformer encoder captures long-range token relations; a multi-head Graph Attention Network (GAT) consumes the encoded sequence together with the adjacency to model explicit molecular topology. Drug-centric Drug Repurposing Knowledge Graph (DRKG) subgraphs are embedded with pretrained TransE and summarized by a lightweight Conv2D → Global Average Pooling (GAP) → Fully Connected (FC) pipeline, producing a size-invariant biological vector. For each pair, we concatenate both drugs' Transformer, GAT, and KG vectors and use a 3-layer FC classifier (Batch Normalization + LeakyReLU) for multi-class DDI.

SSI-DDI — Substructure–substructure interaction without KG.

A molecular graph encoder (GATConv followed by Self-Attention Graph Pooling, SAGPooling) extracts drug-level graph features. A co-attention module estimates fragment × fragment correspondences across the two drugs, and RESCAL-style bilinear scoring yields relation-specific interaction scores. The approach emphasizes fragment interactions and salient subgraphs but does not incorporate external biological knowledge (pathways/targets/diseases).

GMPNN-CS — Relation-aware message passing (size-adaptive).

A message-passing network performs size-adaptive propagation with relation-aware attention: messages depend on node features and learned relation embeddings. Repeated propagation yields drug embeddings that are combined for classification. The method is strong at capturing detailed chemical topology but lacks an external KG, so biology-driven distinctions between similarly structured drugs may be under-modeled.

MUFFIN — CNN over SMILES + TransE KG, with early/elementwise fusion.

Pretrained structure embeddings (GIN-based) and TransE entity/relation embeddings are processed by small Conv2D blocks and global statistics, then fused using early/elementwise rules (e.g., init_double/sum/concat) and fed to a large Multi-Layer Perceptron (MLP). MUFFIN benefits from KG but treats SMILES primarily as sequences for CNNs, so explicit graph topology (rings/branches/bond order) is not modeled.

CASTER / DDE — Chemical substructure fingerprints with AE/Dictionary and a shallow predictor.

Both CASTER and DDE take as input chemical substructure fingerprints, i.e., high-dimensional binary vectors that indicate the presence of molecular fragments. These vectors are compressed by an AutoEncoder (AE) or dictionary encoder; the resulting codes are scaled and passed to a shallow MLP predictor for 86-class DDI. Without explicit topology or KG and with limited classifier depth, the approach struggles to represent complex interaction mechanisms.

2.2 Training hyperparameters

- **TRACE-DDI (ours)**

• Optimizer: Adam; LR = 7.6e-4; Epochs = 100; Batch = 32; Weight Decay: —
• Transformer: d_model = 128; GAT: 12 heads; GAT dropout = 0.0145
• Classifier: FF = 512; FC hidden = 256; Early stop patience = 10
• Positional information: used to preserve order-aware SMILES-derived token representations for the downstream token-to-graph and GAT-based encoding pipeline

- **SSI-DDI**

• Adam; LR = 1e-2; Weight Decay = 5e-4; Epochs = 300; Batch = 1024
• Hidden(atom) = 64; kge_dim = 64; negative = 1; LambdaLR (0.96^epoch)

- **GMPNN-CS**

• Adam; LR = 1e-3; Weight Decay = 5e-4; Epochs = 100; Batch = 512
• Hidden = 64; n_iter = 100; Dropout = 0.0; LambdaLR (0.96^epoch)

- **MUFFIN**

• Adam; LR = 1e-4; Epochs = 200 (early stop = 10); Batch = 2048
• MLP 2048–2048; entity/relation = 100D; structure = 300D

- **CASTER**

• Adam; LR = 1e-3; Epochs = 100; Batch = 256
• AE 1722 → 50; predictor = 1024; λ1 = 1e-2; λ2 = 1e-1; λ3 = 1e-5; magnify = 100

2.3 Architecture comparison

- **TRACE-DDI (ours)**

• Representation: SMILES + adjacency + DRKG
• Topology: ✓ (GAT multi-head)
• Biological: ✓ (Conv2D → GAP KG summary)
• Fusion: Late concat (two drugs × three views)

- **SSI-DDI**

• Representation: Graph (substructure)
• Topology: ✓ (GAT + SAGPooling)
• Biological: ✗
• Fusion: Co-Attention + RESCAL

- **GMPNN-CS**

• Representation: Graph (size-adaptive)
• Topology: ✓ (relation-aware message passing)
• Biological: △ (internal relation embeddings only)
• Fusion: Pair concat

- **MUFFIN**

• Representation: Sequence/Embedding (CNN) + TransE
• Topology: ✗ (explicit topology not modeled)

• Biological: ✓ (TransE 100D)
• Fusion: init_double / sum / concat

- **CASTER / DDE**

• Representation: Chemical substructure fingerprints
• Topology: ✗
• Biological: ✗
• Fusion: —

2.4 Discussion

- **CASTER / DDE (lowest).** Both models rely on chemical substructure fingerprints with an autoencoder/dictionary pipeline, omitting explicit molecular topology and external biological context. They cannot capture non-linear structural phenomena (rings/branches/aromaticity) or pathway-level mechanisms, and their shallow predictors limit capacity for complex 86-class DDI; hence the bottom-tier performance, consistent with the “fingerprint-only” category highlighted in the Introduction.
- **SSI-DDI.** Co-attention and GAT/SAGPooling emphasize fragment-level interactions and reduce noise via pooling, but the absence of KG prevents modeling network-mediated effects (pathways/targets/diseases), limiting discrimination compared with KG-aware approaches.
- **MUFFIN.** Adding KG improves over SSI-DDI, yet processing SMILES with CNN/Conv2D omits explicit topology and fusion is primarily early/elementwise, constraining high-order structure × biology interactions—therefore below our hybrid.
- **GMPNN-CS.** Relation-aware message passing excels at capturing chemical topology, but without an external KG it struggles to separate biologically distinct pairs sharing similar structural motifs.
- **TRACE-DDI (ours).** Combining long-range sequence semantics (Transformer), local topology (GAT), and size-invariant KG pooling (Conv2D → GAP) with late fusion enables complementary, high-order interactions, yielding top accuracy and F1.

2.5 Reproducibility and fairness

For all baselines, we retained their default training/classification hyperparameters from the source code and only adapted minimal preprocessing to align input formats with our dataset. Every model used the same stratified 5-fold protocol as in the main paper. Seeds followed each project’s defaults; other models used their code defaults. These controls support a fair architectural comparison, indicating that performance differences primarily reflect representational capacity rather than retuning advantages.

3. Extended Visualizations of Anchor–Pathway Relationships Across Evaluated Drug–Drug Interaction Labels

In the main manuscript, we reported only two representative anchor–pathway examples for clarity. In this Supplementary section, we provide additional visualizations generated by the same centrality-based analysis pipeline in order to illustrate how the framework extends across multiple anchors and interaction labels.

For each anchor drug A and interaction label y, we first identified pathway candidates X that were (i) present in the anchor-centered subgraph and (ii) shared by at least a minimum fraction of interacting partner drugs under label y. For each retained candidate, pathway centrality values were measured within merged drug-pair subgraphs and compared between interacting and non-interacting partner groups. Candidate pathways were then ranked using a composite score based on centrality separation, eigenvector centrality among interacting partners, and pathway coverage ratio.

Because the strict screening criterion did not retain stable candidates in the current dataset, the supplementary examples shown here were selected from the relaxed screening stage of the pipeline. These examples should therefore be interpreted as exploratory, hypothesis-generating visualizations rather than confirmatory evidence.

We provide two complementary visualization modalities. First, centrality distribution plots (KDE) compare the distributions of pathway centrality values in merged subgraphs of interacting and non-interacting partner groups. The vertical dashed line indicates the 95th percentile threshold, and rug marks highlight observations above this cutoff. These plots provide an intuitive view of whether a pathway tends to occupy a more topologically prominent position in interacting contexts. Second, merged subgraph diagrams display a representative interacting partner B together with anchor drug A, while highlighting pathway X. Node colors indicate provenance (anchor-specific, partner-specific, or overlapping nodes), and the highlighted pathway is emphasized as the focal mediator. Centrality values for the highlighted pathway are summarized within the panel.

Together, these supplementary figures extend the pathway-level interpretation presented in the main manuscript and illustrate the consistency of the proposed analysis framework across multiple drug–drug interaction settings.

Case-study selection pipeline.

To improve reproducibility and clarify how representative examples were selected, we summarize the quantitative case-study selection procedure used in this study. For each anchor drug and interaction label, we first defined interacting and non-interacting partner groups from the DDI table. We then identified pathway candidates shared between the anchor-centered subgraph and partner-centered subgraphs, retaining only those that satisfied a minimum coverage-ratio threshold across interacting partners. For each retained pathway candidate, pathway centrality values were measured in merged drug-pair subgraphs for both interacting and non-interacting partner groups. The candidates were then ranked using a composite score based on centrality separation, eigenvector prominence in the interacting group, and pathway coverage. Finally, the ranked candidates were screened using Mann–Whitney U statistics, Benjamini–Hochberg false-discovery-rate correction, Cliff's delta, and consistency of positive effect direction across multiple centrality measures. The main-text case studies were therefore selected from a predefined quantitative screening pipeline rather than by retrospective narrative choice, and should be interpreted as hypothesis-generating examples rather than confirmatory findings.

Algorithm 1 Case-study selection pipeline

```
Input: DDI table, anchor drugs, interaction labels, drug-centered subgraphs
Output: Representative case-study candidates
for each anchor drug A do
  for each interaction label y do
    Construct interacting and non-interacting partner sets
    Filter out groups below the minimum size threshold
    Identify shared pathway candidates
    Apply minimum coverage-ratio filtering
    for each retained pathway candidate X do
      Compute centrality values in interacting merged subgraphs
      Compute centrality values in non-interacting merged subgraphs
      Compute separation statistics
      Compute composite ranking score
    end for
    Rank pathway candidates for (A, y)
    Retain top candidates
  end for
end for
Pool candidates across anchors and labels
Apply statistical screening
Select representative case studies
Return final candidates
```

3.1 Representative exploratory signals from the relaxed screening stage

In addition to the representative visual examples shown below, we summarize the anchor–interaction–pathway combinations that were retained by the relaxed screening stage of our analysis pipeline. These candidates did not satisfy the stricter multi-metric screening criterion, but they showed at least one favorable centrality signal under the relaxed rule, which combined nominal significance, positive mean separation, and a minimum effect-size threshold.
Accordingly, the signals listed in Table S2 should be interpreted as exploratory candidates used to support supplementary visualization and qualitative inspection. They are not presented as confirmatory pathway discoveries, but rather as

hypothesis-generating examples that illustrate how the pathway-centered analysis framework can identify potentially informative mediator structures across different anchor drugs and interaction labels.

Interaction label indices correspond to the definitions listed in Supplementary Table S1.

Table S2. Representative exploratory anchor–interaction–pathway signals selected from the relaxed screening stage. These candidates were used as supplementary visualization examples because no pathway satisfied the stricter multi-metric screening criterion in the current dataset. “Best-supported metric” denotes the metric showing the strongest favorable signal among the relaxed candidates.

Anchor	Interaction label	Pathway	n_int	n_non	Coverage ratio	Best-supported metric	q-value	Δmean	Cliff’s delta	Selection note
541	58	Pathway::PC7_8339	8	403	0.1860	Degree	0.0139	0.0054	0.4544	Relaxed screening
76	49	Pathway::PC7_3993	5	125	0.1923	Eigenvector	0.0666	0.0239	0.3984	Relaxed screening
571	55	Pathway::PC7_3278	5	391	0.1563	Degree	0.0351	0.0055	0.4716	Relaxed screening
1388	44	Pathway::PC7_5322	12	680	0.3636	Degree	0.0285	0.0048	0.3202	Relaxed screening
340	14	Pathway::PC7_4112	55	295	0.2764	Closeness	1.30e-09	0.0144	0.5057	Relaxed screening
571	55	Pathway::PC7_5887	6	228	0.1875	Degree	0.0450	0.0046	0.4064	Relaxed screening
340	14	Pathway::WP2377_r80437	25	69	0.1256	Closeness	0.0081	0.0072	0.3264	Relaxed screening

Interpretation.

As shown in Table S2, the retained exploratory candidates typically exhibited modest but positive separation in at least one centrality metric, together with non-trivial coverage across interacting partner drugs. In particular, some candidates were preferentially supported by degree centrality, whereas others were more strongly supported by closeness or eigenvector centrality. This pattern suggests that pathway prominence may emerge through different topological roles depending on the anchor drug and interaction label. Because these examples originate from the relaxed screening stage, they are used here to motivate supplementary visual inspection rather than to establish formal statistical confirmation.

3.2 Supplementary KDE and merged-subgraph visualizations

The following figures present selected supplementary examples corresponding to the exploratory candidates summarized above. For each example, we provide a KDE-based comparison of pathway centrality distributions between interacting and non-interacting partner groups, together with a merged subgraph visualization highlighting the corresponding pathway within a representative drug-pair context. These panels are intended to complement the main-text case studies by showing that the same analysis framework can be extended to additional anchor–interaction settings.

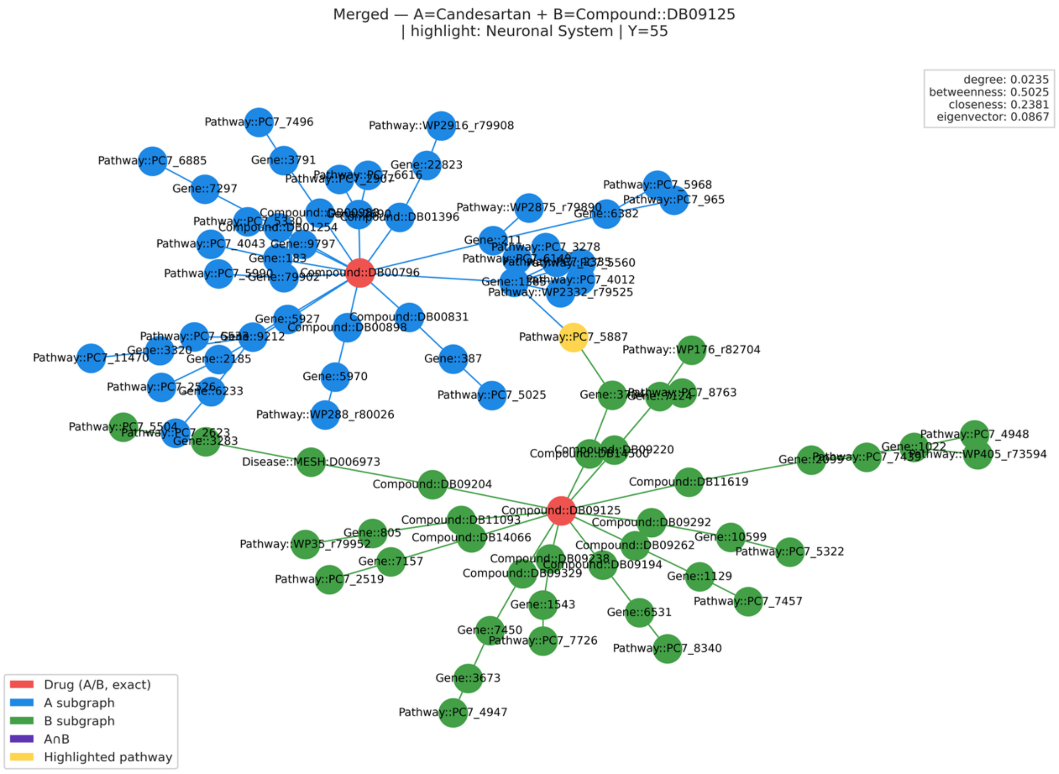

Merged — A=Candesartan + B=Compound::DB09125
| highlight: Neuronal System | Y=55
degree: 0.0235
betweenness: 0.5025
closeness: 0.2381
eigenvector: 0.0867
Pathway::PC7_7496
Pathway::WP2916_r79908
Pathway::PC7_6885
Gene::3791
Gene::22823
Gene::7297
Compound::DB01396
Pathway::WP2875_r79890
Pathway::PC7_5968
Pathway::PC7_965
Gene::6382
Compound::DB01254
Pathway::PC7_4043
Gene::9797
Gene::183
Pathway::PC7_5990
Compound::DB00796
Pathway::PC7_3278
Pathway::PC7_4012
Pathway::WP2332_r79525
Gene::5927
Compound::DB00831
Compound::DB00898
Pathway::PC7_5887
Pathway::WP176_r82704
Pathway::PC7_11470
Gene::2185
Gene::387
Gene::5970
Pathway::PC7_5025
Gene::6233
Pathway::PC7_8763
Pathway::WP288_r80026
Pathway::PC7_5504
Gene::3283
Pathway::PC7_4948
Pathway::WP405_r73594
Disease::MESH:D006973
Compound::DB09220
Compound::DB11619
Compound::DB09204
Compound::DB09125
Compound::DB11093
Compound::DB09292
Compound::DB14066
Gene::10599
Pathway::PC7_5322
Pathway::WP35_r79952
Gene::805
Gene::7157
Compound::DB09262
Compound::DB09194
Compound::DB09329
Gene::1129
Pathway::PC7_7457
Pathway::PC7_2519
Gene::1543
Gene::6531
Gene::7450
Pathway::PC7_7726
Pathway::PC7_8340
Gene::3673
Pathway::PC7_4947
Drug (A/B, exact)
A subgraph
B subgraph
A∩B
Highlighted pathway

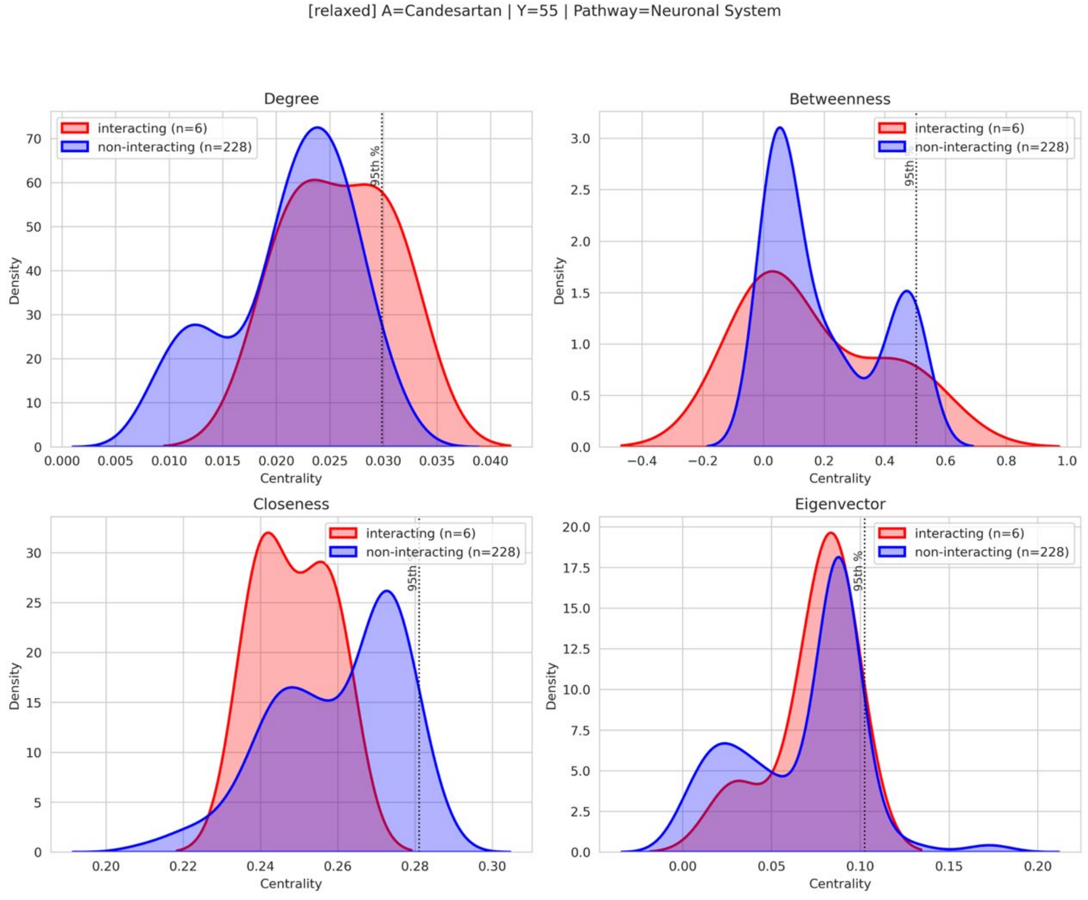

[relaxed] A=Candesartan | Y=55 | Pathway=Neuronal System
Degree
Betweenness
Closeness
Eigenvector
interacting (n=6)
non-interacting (n=228)
95th %
Density
Centrality

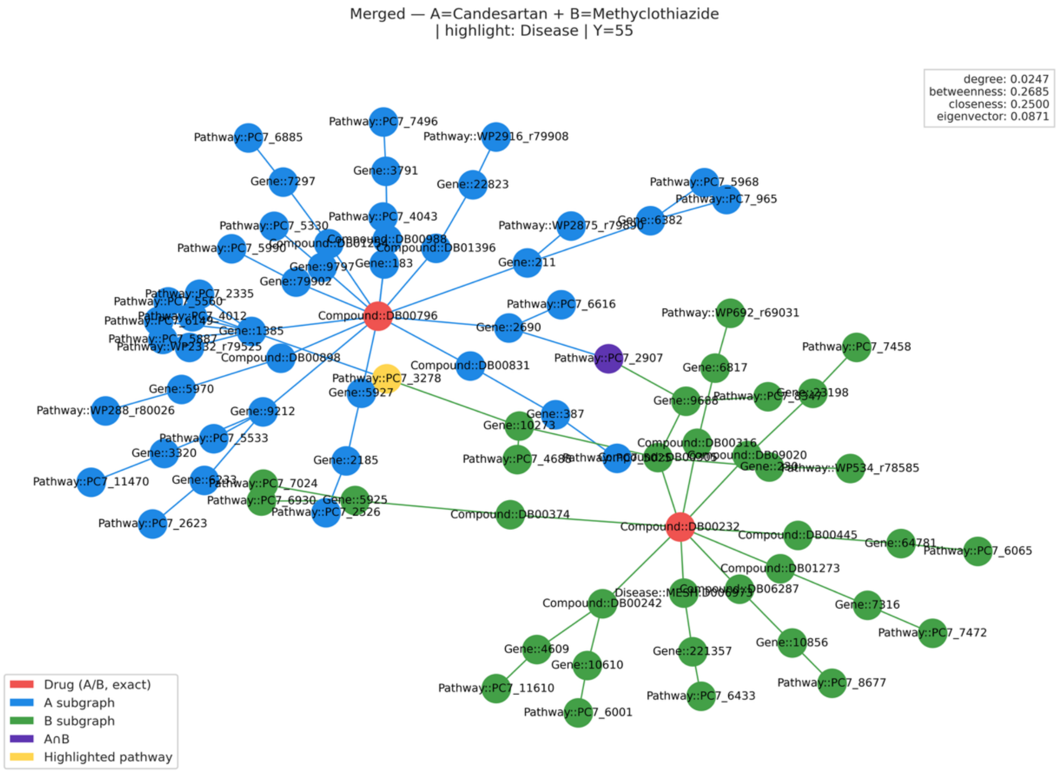
Merged — A=Candesartan + B=Methyclothiazide
| highlight: Disease | Y=55
degree: 0.0247
betweenness: 0.2685
closeness: 0.2500
eigenvector: 0.0871
Compound::DB00796
Compound::DB00232
Pathway::PC7_3278
Pathway::PC7_2907
Drug (A/B, exact)
A subgraph
B subgraph
A∩B
Highlighted pathway

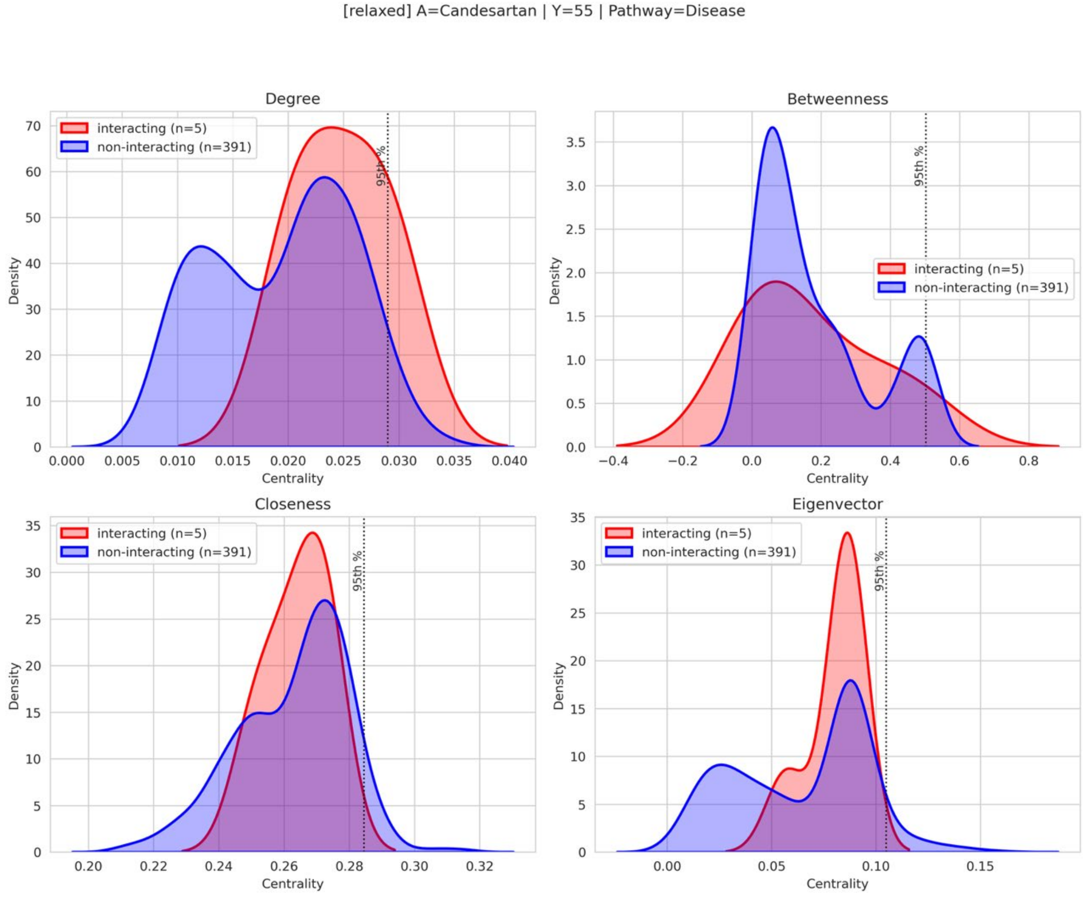
[relaxed] A=Candesartan | Y=55 | Pathway=Disease
Degree
Betweenness
Closeness
Eigenvector
interacting (n=5)
non-interacting (n=391)
95th %
Density
Centrality

4. Random-Walk Hyperparameter Sensitivity Check

Experimental setting

To assess whether pathway reachability in the preprocessing stage was overly sensitive to the random-walk hyperparameters, we performed a limited sensitivity check using representative alternative settings. We compared three configurations that varied the effective sampling budget through either the number of iterations or the walk length, while keeping the restart probability fixed. For each configuration, we recorded the number of compounds that did not reach any pathway node during random-walk preprocessing.

Table S3. Limited sensitivity check for random-walk preprocessing.

iteration	Steps	Unreachable compounds
2	2000	92
100	2000	6
2	20000	6

The table reports the number of compounds that did not reach any pathway node under representative random-walk settings.

Interpretation

Under a low-budget setting (iteration = 2, steps = 2000), the number of pathway-unreachable compounds was relatively high (92). However, when the sampling budget was increased either by raising the number of iterations (iteration = 100, steps = 2000) or by increasing the walk length (iteration = 2, steps = 20000), the number of unreachable compounds decreased to 6 in both cases. This pattern suggests that pathway reachability becomes substantially more stable once sufficient sampling effort is provided, and that the adopted random-walk configuration reflects a practical choice for robust pathway-connected subgraph extraction rather than an arbitrary parameter setting. Because this check was designed as a limited representative comparison rather than an exhaustive sensitivity analysis across all parameter combinations, broader parameter sweeps remain an important direction for future work.

5. Primary 86-Class Pair-Level Benchmark: Overlap and Fold-wise Performance

5.1 Drug overlap across folds

Table S4. Fold-wise drug overlap in the 86-class pair-level benchmark.

Fold	Train drugs	Validation drugs	Shared drugs	Shared / Validation (%)
1	1672	1605	1586	98.8
2	1683	1586	1578	99.5
3	1676	1590	1575	99.1
4	1681	1590	1580	99.4
5	1674	1593	1576	98.9

Table S4 summarizes fold-wise drug overlap statistics in the primary 86-class pair-level cross-validation benchmark. Because splitting is performed at the pair level, nearly all validation drugs are also present in the corresponding training folds, confirming that this benchmark should be interpreted as a transductive evaluation rather than a strict unseen-drug setting.

5.2 Fold-wise overall performance

Table S5. Fold-wise overall performance in the 86-class multi-class benchmark.

Fold	Accuracy	Weighted Precision	Weighted Recall	Weighted F1	Macro Precision	Macro Recall	Macro F1
1	0.9737	0.9737	0.9737	0.9736	0.9705	0.9436	0.9472
2	0.9729	0.9732	0.9729	0.9729	0.9516	0.9334	0.9336
3	0.9740	0.9742	0.9740	0.9740	0.9468	0.9480	0.9439
4	0.9745	0.9746	0.9745	0.9745	0.9597	0.9554	0.9553
5	0.9745	0.9745	0.9745	0.9745	0.9582	0.9469	0.9499
Mean	0.9739	0.9740	0.9739	0.9739	0.9574	0.9455	0.9460

Table S5 reports fold-wise overall performance of TRACE-DDI in the primary 86-class multi-class benchmark. Weighted metrics remain consistently high across folds, whereas macro-averaged metrics are lower, reflecting the increased difficulty of minority interaction types under the long-tail label distribution.

5.3 Macro and class-wise performance example

To complement the fold-wise averaged benchmark, we additionally provide the detailed classification report from the best-score run of the primary 86-class setting. This result is included to illustrate macro-averaged behavior and per-class variation more explicitly. Because it corresponds to a single run rather than the cross-fold mean, it should be interpreted as an illustrative example rather than as the primary summary statistic of benchmark performance.

Table S6. Overall metrics of the best-score run in the primary 86-class benchmark.

Metric	Value
Accuracy	0.9755
Weighted Precision	0.9756
Weighted Recall	0.9755
Weighted F1	0.9755
Macro Precision	0.9695
Macro Recall	0.9440
Macro F1	0.9439

Table S7. Selected class-wise results from the best-score run in the primary 86-class benchmark.

Class	Precision	Recall	F1-score	Support
1	0.9701	1.0000	0.9848	65
3	0.9408	0.9656	0.9530	988
5	0.9858	0.9873	0.9865	631
15	0.9899	0.9954	0.9927	1086
19	0.9528	0.9730	0.9628	1224
46	0.9660	0.9774	0.9717	6829
48	0.9907	0.9849	0.9878	12179
59	0.9857	0.9827	0.9842	1679
69	0.9824	0.9685	0.9754	1554
72	0.9594	0.9537	0.9565	4708
74	0.9557	0.9352	0.9453	1867
76	0.9259	0.9259	0.9259	108
81	0.9531	0.8714	0.9104	70
84	0.9367	1.0000	0.9673	74

As shown in Tables S5 and S6, the best-score run exhibits the same overall tendency as the fold-wise average benchmark: weighted metrics remain very high, whereas macro-averaged metrics are lower because minority classes remain more

challenging. High-support classes are predicted consistently well, while a subset of lower-support classes still shows performance instability. This pattern supports the interpretation that TRACE-DDI performs strongly under the transductive pair-level benchmark, while class imbalance continues to affect minority interaction categories.

5.4 Interpretation

The fold-wise drug overlap statistics show that the primary 86-class benchmark is strongly transductive in nature. Because almost all validation drugs are also observed in the corresponding training folds, this evaluation primarily measures generalization to new drug pairs formed from largely previously observed drugs, rather than generalization to entirely unseen compounds.

At the same time, the fold-wise performance results remain informative for the original multi-class benchmark because pair-level stratified splitting preserves class support under the pronounced long-tail label distribution. In particular, several rare interaction types have very limited samples, and a strict drug-level split would cause some classes to disappear from either the training or validation folds. Thus, the pair-level benchmark is retained as the primary evaluation protocol for stable 86-class learning, while the strict unseen-drug experiment in Section 6 serves as a complementary inductive stress test.

Importantly, the consistent gap between weighted and macro-averaged metrics indicates that TRACE-DDI performs strongly overall, but minority interaction types remain more challenging than common classes. Therefore, the reported multi-class results should be interpreted as strong transductive benchmark performance rather than as evidence of fully inductive unseen-drug generalization.

6. Strict Unseen-Drug Evaluation

6.1 Experimental setup

To evaluate inductive generalization to previously unseen drugs, we conducted a strict drug-holdout experiment under a binary DDI prediction setting. The complete drug set was randomly partitioned into “seen” and “holdout” subsets. Approximately 20% of drugs were assigned to the holdout set. The model was trained exclusively on pairs composed of seen drugs (seen–seen pairs) and evaluated only on pairs composed of holdout drugs (holdout–holdout pairs). Pairs spanning the two subsets were excluded to prevent cross-set information sharing.

6.2 Dataset statistics and overall results

Table S8. Summary of the strict unseen-drug binary evaluation. The complete drug set was partitioned into disjoint seen and holdout subsets. Training used only seen–seen pairs, testing used only holdout–holdout pairs, and mixed seen–holdout pairs were excluded.

Item	Value
Holdout fraction	0.20
Holdout drugs	338
Shared drugs (train ∩ test)	0
Dropped cross pairs	123,225
Evaluation pairs	15,517
Accuracy	0.5990
ROC-AUC	0.6541
PR-AUC	0.6725

Item	Value
Macro F1	0.5828
Weighted F1	0.5803

This strict drug-holdout setting constitutes a fully inductive cold-start evaluation because neither drug in the test pairs is observed during training.

6.3 Class-wise results

Table S9. Class-wise performance in the strict unseen-drug binary evaluation. Class 0 denotes non-interacting pairs and class 1 denotes interacting pairs.

Class	Precision	Recall	F1-score	Support
0	0.5589	0.8207	0.6649	7,524
1	0.6981	0.3902	0.5006	7,993
Macro avg	0.6285	0.6055	0.5828	15,517
Weighted avg	0.6306	0.5990	0.5803	15,517

6.4 Interpretation

The strict unseen-drug binary evaluation is intentionally much more difficult than the primary 86-class pair-level benchmark. In this setting, neither drug in the test pairs is observed during training, and mixed seen–holdout pairs are removed entirely. The resulting performance is therefore expected to be substantially lower than in the transductive pair-level benchmark.

Nevertheless, TRACE-DDI retains non-trivial discriminative ability under this fully inductive protocol, with ROC-AUC of 0.6541 and PR-AUC of 0.6725. These results indicate that the model does not rely solely on memorization of previously observed drug identities or pair co-occurrence patterns.

Importantly, this experiment reused the same core TRACE-DDI architecture and nearly the same training configuration as the original 86-class model. The only architectural modification was replacing the final output layer with a two-class classifier, and no binary-specific hyperparameter tuning was performed. Accordingly, these results should be interpreted as a conservative estimate of unseen-drug generalization rather than as the best achievable performance under a binary-optimized setting.

The class-wise results further show that the positive interaction class is more difficult to recover in the strict unseen-drug setting, as reflected by the relatively low recall for class 1. This asymmetry is consistent with the increased difficulty of predicting true DDIs when both compounds are entirely unseen during training.

7. RDKit-Based Graph Validation

7.1 Experimental setup.

To assess whether TRACE-DDI depends critically on the custom SMILES-to-graph parser, we conducted an additional validation experiment using RDKit-derived molecular graphs. In this setting, adjacency matrices and bond information were generated directly from RDKit, while all other components of TRACE-DDI—including the Transformer encoder, GAT module, KG embeddings, and classifier—were kept unchanged.

7.2 Results.

The RDKit-based variant achieved the following overall performance:

Metric	Value
Accuracy	0.9538
Weighted Precision	0.9538
Weighted Recall	0.9538
Weighted F1	0.9537
Macro Precision	0.9332
Macro Recall	0.9005
Macro F1	0.8991

7.3 Interpretation.

These results indicate that TRACE-DDI remains effective under a standardized cheminformatics graph-construction pipeline. Although the RDKit-based setting underperformed relative to the custom graph-construction pipeline used in the main experiments, its performance remained strong overall. This suggests that the reported performance of TRACE-DDI is not solely dependent on parser-specific artifacts, while also indicating that graph-construction details influence downstream predictive quality.

8. Comparison of KG Aggregation Methods

8.1 Experimental setting

To examine the effect of the KG aggregation strategy more directly, we compared four alternatives for summarizing the DRKG-derived subgraph embeddings: Conv-based aggregation, mean pooling, sum pooling, and PCA-based reduction. In this experiment, all other components of TRACE-DDI were kept unchanged.

To focus on relative differences among aggregation methods under a shared comparison setting, all variants were evaluated using the same training configuration rather than separately re-optimizing hyperparameters for each method. The shared configuration was as follows: 5-fold multiclass cross-validation, 100 epochs, batch size = 32, learning rate = 0.00076, Transformer hidden dimension = 128, embedding dimension = 64, number of Transformer heads = 4, number of Transformer encoder layers = 3, feedforward dimension = 512, classifier hidden dimension = 256, classifier dropout = 0.0, GAT dropout = 0.0145, GAT alpha = 0.3086, and number of GAT heads = 8.

Accordingly, this experiment should be interpreted as a controlled relative comparison of KG aggregation strategies under a shared configuration, rather than as a comparison of separately optimized model variants.

8.2 Comparative results and brief interpretation

The aggregation methods showed measurable but relatively modest performance differences under this shared setting. The Conv-based aggregation remained highly competitive, showing performance very close to mean pooling and outperforming PCA-based reduction and sum pooling. These results indicate that the choice of KG aggregation method affects downstream DDI prediction performance, while also showing that the performance gap between Conv-based aggregation and mean pooling is small under this shared comparison setting. Importantly, the Conv-based module was retained in the main TRACE-DDI framework based on its modeling rationale and its competitive performance under our comparison setting, rather than being interpreted as the only viable aggregation strategy. Specifically, the Conv-based design was introduced to provide a learnable aggregation mechanism for variable-sized KG subgraphs composed of heterogeneous entity and relation embeddings, while simultaneously performing dimensional compression into a fixed-length drug representation. In this sense, the Conv block was intended as a practical nonlinear set-to-vector projection module that can flexibly summarize diverse subgraph patterns, rather than as a biologically spatial operator. The present supplementary comparison therefore serves to show that the Conv-based design remains competitive under a shared comparison setting, while simpler fixed reductions can also perform strongly.

Table S10. Comparison of KG aggregation methods under a controlled shared training configuration. All variants were evaluated using the same shared training configuration without separate hyperparameter re-optimization for each aggregation method.

KG aggregation method	Accuracy	Weighted Precision	Weighted Recall	Weighted F1	Macro Precision	Macro Recall	Macro F1
Conv	0.9491	0.9499	0.9491	0.9492	0.9176	0.9259	0.9146
Mean	0.9499	0.9500	0.9499	0.9496	0.9322	0.9181	0.9197
PCA	0.9416	0.9419	0.9416	0.9415	0.9167	0.9182	0.9128
Sum	0.9369	0.9374	0.9369	0.9367	0.9274	0.8962	0.8979

Among the compared KG aggregation methods under this controlled shared training configuration, the Conv-based aggregation remained highly competitive, showing performance very close to mean pooling while outperforming PCA-based reduction and sum pooling. These results indicate that the choice of KG aggregation method affects downstream performance, while also suggesting that simpler fixed reductions can perform strongly under a shared configuration.

This shared comparison configuration was used only for the supplementary KG aggregation comparison and is distinct from the main TRACE-DDI configuration.

9. Effect of Positional Information in the Token-to-Graph Encoding Pipeline

9.1 Experimental setting

To evaluate the contribution of positional information in TRACE-DDI, we conducted an ablation experiment in which the positional signal applied to SMILES-derived token representations was removed while keeping the remaining architecture unchanged. In our framework, this positional information is not introduced merely as a generic component of Transformer sequence modeling. Rather, it is used to preserve structurally distinguishable token representations that are subsequently used in the downstream token-to-graph construction and GAT-based encoding process.

Accordingly, this experiment was designed to assess the extent to which positional information contributes to maintaining an order-aware structural basis for molecular graph construction and graph representation learning in TRACE-DDI.

9.2 Comparative results and brief interpretation

Removing positional information caused a severe collapse in predictive performance. In the ablated setting, overall accuracy dropped to 0.5159 and weighted F1 to 0.4800, while macro F1 decreased sharply to 0.1774. This degradation indicates that, without positional information, TRACE-DDI fails to preserve sufficiently structured and distinguishable SMILES-derived token representations for the downstream token-to-graph and GAT-based encoding pipeline.

The deterioration was especially pronounced at the class-wise level, where many interaction types showed near-zero recall, indicating that the model lost much of its ability to maintain discriminative structure across the multi-class prediction task. These results support our design choice that positional information in TRACE-DDI is critical not merely for sequence

modeling, but for preserving an order-aware structural basis for downstream graph construction and topology-aware molecular representation learning.
